# Clonal dynamics shaped by diverse drug-tolerant persister states in melanoma resistance

**DOI:** 10.1101/2025.09.16.676608

**Published:** 2025-09-19

**Authors:** Haiyin Li, Yeqing Chen, Jessica Kaster, Maggie Dunne, Min Xiao, Ling Li, Monzy Thomas, Nazifa Promi, Dylan Fingerman, Gregory Schuyler Brown, Qiuxian Zheng, Xingyue Zhu, McKenna Reale, Andrew Patterson, Le Gao, Xuxiang Zhang, Siqi Jiang, Tianxing Hu, Hanzhang Fang, Jianlan Ren, Cong Qi, Luyang Wang, Haiwei Mou, Gatha Thacker, Eric Ramirez Salazar, Jessie Villanueva, Arjun Raj, Dave SB Hoon, Tian Bin, Jozef Madzo, Zhi Wei, Noam Auslander, Meenhard Herlyn

**Affiliations:** 1 The Wistar Institute, Philadelphia, PA, USA; 2 Department of Computer Science, New Jersey Institute of Technology, Newark, NJ, USA; 3 Department of Bioengineering, School of Engineering and Applied Sciences, University of Pennsylvania, Philadelphia, PA, USA; 4 Department of Genetics, Perelman School of Medicine, University of Pennsylvania, Philadelphia, PA, USA; 5 St John’s Cancer Institute, Santa Monica, CA, USA

## Abstract

Most advanced melanomas initially respond to targeted therapy but eventually relapse. Rather than acquiring new mutations, resistance is driven by drug-tolerant persister cells that enter a reversible drug-refractory state. We developed MeRLin, a high-resolution lineage tracing platform integrating cellular barcoding, single-cell transcriptomics, RNA fluorescence in situ hybridization (FISH), and computational analyses to track clonal and transcriptional dynamics in patient-derived melanoma models during prolonged therapy. Clonal dynamics revealed that persister subpopulations first responded to treatment but persisted and expanded during minimal residual disease, ultimately leading to tumor recurrence. Pre-treatment melanoma populations diversified into four conserved persister states characterized by stress-like, lipid metabolism, PI3K signaling, and extracellular matrix remodeling programs associated with adaptive resistance. Spatial transcriptomics showed the organization of these adaptive programs and a complex signaling network of autocrine and paracrine interactions among persister subpopulations. Barcoded RNA-FISH enabled spatial mapping of clonal identity and gene expression, revealing *in situ* co-localization of a dominant resistant clone with *SLC2A1* expression. MeRLin provides a robust framework for dissecting cancer heterogeneity and identifying vulnerabilities in persister populations.

## Introduction

Despite initially exhibiting strong responses to targeted therapy, the majority of tumors eventually relapse, often without gaining new resistance mutations^1,2^. The presence of drug-tolerant persister (DTP) cells, a subpopulation of cancer cells that enter a reversible drug-refractory state, contributes to minimal residual disease (MRD)^3^. DTP-mediated resistance stems from intratumoral phenotypic heterogeneity driven by diverse gene expression programs and adaptive survival mechanisms^3^. During treatment, cancer cells may transiently activate survival pathways and resistance genes, entering a DTP state^3^. Prolonged drug exposure enables DTP cells to develop diverse, heritable resistance, allowing them to tolerate cytotoxic stress, establish MRD, and seed subsequent outgrowth of resistant clones^4^. It remains unclear how distinct DTP states emerge from an initially homogeneous population. Understanding the mechanisms of DTP formation, plasticity, and heterogeneity in cancer is crucial for the discovery of resistance markers and therapeutic targets^3^. The detection and targeting of DTP cells offer novel strategies to overcome resistance and improve long-term treatment efficacy^1,5^.

Here, we developed MeRLin (Melanoma Resistance Lineage tracing), a lineage tracing technology that uniquely integrates single-cell RNA sequencing (scRNA-seq), RNA fluorescence *in situ* hybridization (RNA-FISH), and computational analyses. By direct retrieval of barcode sequences from mRNAs, MeRLin allows simultaneous clonal tracing and transcriptional profiling at single-cell resolution, along with spatial validation of specific clonal populations. Employing MeRLin in a melanoma model that recapitulates the clinical emergence of BRAF/MEK inhibitor (BRAFi/MEKi) resistance, we traced the clonal origins and transcriptional states of thousands of individual melanoma cells as they acquired resistance *in vivo*. Prolonged targeted therapy led to the emergence of molecularly and functionally diverse DTP states from initially homogeneous, minor pre-treatment subpopulations. The DTP states are marked by four adaptive programs. We identified genes enriched in persister subpopulations and spatially mapped a dominant resistant clone marked by *SLC2A1* expression in the resistant tumor. Overall, MeRLin revealed the key mechanisms, pathways, and genes driving MRD and tumor recurrence, uncovering potential vulnerabilities that could be targeted to delay or prevent drug resistance^6^.

## Results

### Development of MeRLin for single-cell lineage tracing and transcriptomic profiling

**Fig. 1.**
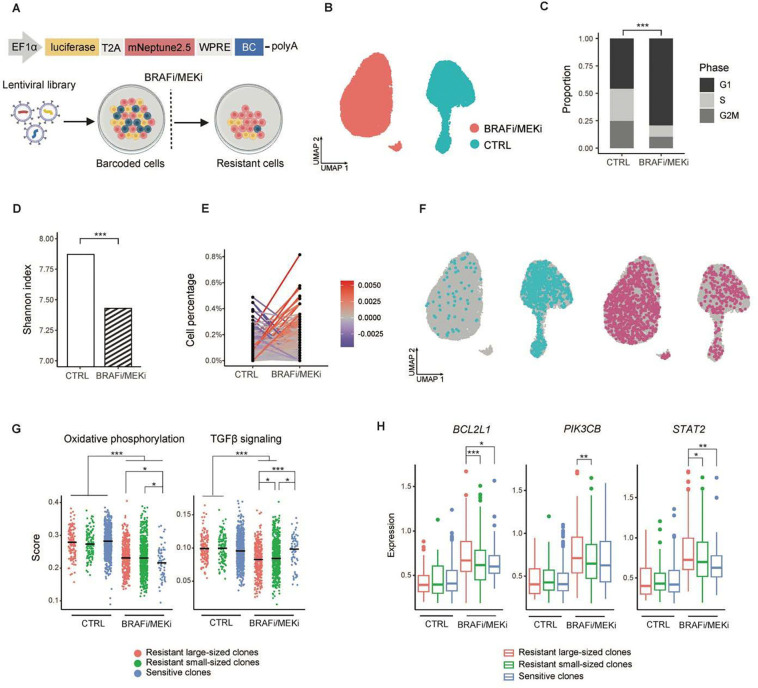
Distinct clone fates are associated with variable expression programs. **a**, Schematic representation of the MeRLin construct and barcoded WM4237–1 cells exposed to targeted therapy^13^. The vector encoded firefly luciferase and fluorescent protein mNeptune2.5. Lineage tracing is enabled by semi-random barcodes (BC) transcribed within the 3’-untranslated region of mRNAs. WM4237–1 cells were transduced with the MeRLin barcode library. EF1α, elongation factor 1α; T2A, self-cleaving peptide; WPRE, woodchuck hepatitis virus post-transcriptional regulatory element; PolyA, polyadenylation signal. **b**, Uniform manifold approximation and projection (UMAP) to visualize the separation of BRAFi/MEKi-treated (red) and control (CTRL, blue) WM4237–1 cells using scRNA-seq data. **c**, Proportion of cells in each cell cycle phase inferred from scRNA-seq. P < 2.2 × 10^−16^, two-tailed Fisher's exact test. **d**, Shannon diversity indices (CTRL vs. BRAFi/MEKi-treated cells). P < 2.2 × 10^−16^, two-tailed Hutcheson’s t-test. **e**, Lineage tracing of BRAFi/MEKi-treated cells to pre-treatment ancestor cells (CTRL), quantified by the percentage of cells carrying each specific barcode. **f**, UMAP showing sensitive clones (blue) and resistant clones (red) following treatment. **g**, Resistant large-sized, resistant small-sized and sensitive clones showed significance in cellular expression programs. And in differentially expressed genes **h,** *P < 0.05, **P < 0.01, ***P < 0.001; two-tailed Wilcoxon rank-sum test.

Phenotypic heterogeneity in cancer cell populations provides the foundation for clonal selection, particularly under drug pressure. Both Darwinian selection of pre-existing resistant clones and Lamarckian induction of drug-tolerant mechanisms can contribute to the emergence of DTP cells^1^. We developed MeRLin, a high-complexity lentiviral barcoding system designed to simultaneously track clonal dynamics and transcriptional states at single-cell resolution (Fig. 1A and Fig. S1A–S1C). The vector backbone encodes firefly luciferase and far-red fluorescent protein mNeptune2.5, enabling *in vivo* imaging (Methods and Fig. S1D). Lineage tracing is achieved by sequencing clone-specific transcribed barcodes within the 3′-untranslated region (UTR) of luciferase and mNeptune2.5 mRNAs (Methods). These barcodes consist of 265 bp semi-random sequences, allowing specific cancer subpopulations to be visualized using targeted RNA fluorescence *in situ* hybridization (RNA-FISH).

We constructed a MeRLin library of 2.89 million barcodes and transduced WM4237–1 melanoma cells with a multiplicity of infection (MOI) less than 0.4 to ensure each cell carried a unique barcode (Methods; Table S2). WM4237–1 was derived from a 29-year-old female patient with a BRAF V600E mutation prior to BRAFi/MEKi treatment^7^ (Table S3). Infected WM4237–1 cells were sorted for mNeptune2.5-positive populations using flow cytometry (Methods). The barcoded populations were expanded and divided into replicates, with one replicate saved as the original control.

### Lineage tracing reveals selection of minor clones with resistant phenotypes

We first analyzed the clonal selection of barcoded WM4237–1 cells under BRAFi/MEKi *in vitro* (Fig. 1A). Cell replicates were treated with 0.3μM dabrafenib and 30 nM trametinib for a week, resulting in 20–30% survival of resistant cells (Methods). We performed scRNA-seq on both the BRAFi/MEKi-treated and control cells (Fig. 1B). The analysis revealed that treated cells exhibited G1 cell cycle arrest (Fig. 1C; Methods) and upregulation of melanocytic markers, including *EDNRB, DCT*, and *TYRP1* (Fig. S2A), indicating a more differentiated transcriptional state^8^. Single-cell regulatory network inference and clustering (SCENIC) was employed to infer gene regulatory networks from the scRNA-seq data (Methods; Fig. S2B). In control cells, we observed high *MYC* activity, a driver of epithelial-mesenchymal transition (EMT), and poor prognosis in melanoma^9^, while *JUN* regulatory activity was increased in treated cells, promoting early adaptive EMT-like changes during BRAF inhibitor treatment^10^ (Fig. S2C).

Barcode reads were directly retrieved from scRNA-seq data, enabling the tracking of treated cells back to their pre-treatment ancestors in the original control (Methods). This approach allowed simultaneous lineage tracing and comparison of transcriptional states between treated and control cells. Following treatment, barcode diversity was markedly reduced, with less than 25% of the control cells surviving BRAFi/MEKi pressure (Fig. S2D). Shannon index measuring barcode richness and evenness (Methods) revealed a moderate decrease in barcode diversity in treated cells compared to pre-treatment samples (Fig. 1D). The dominant resistant clones originated from minor pre-treatment subpopulations (Fig. 1E). Sensitive clones were eliminated upon BRAFi/MEKi treatment (Fig. 1F, left), whereas resistant clones emerged after treatment (Fig. 1F, right).

To identify the cellular expression programs associated with resistant clones, we analyzed gene sets that were differentially expressed in these cells (Methods). We found that oxidative phosphorylation (OXPHOS) contributes to the metabolic plasticity that underlies melanoma resistance (Fig. 1G), consistent with previous reports^11^. Sensitive clones showed the greatest reduction in OXPHOS from pre- to post-treatment, while resistant clones maintained higher OXPHOS levels compared to sensitive clones. Moreover, TGFβ (transforming growth factor β) signaling was downregulated in resistant clones following BRAF/MEK inhibition (Fig. 1G), whereas sensitive clones retained active TGFβ signaling, which may promote apoptosis when combined with MAPK pathway inhibition^12^. The resistant clones expressed distinct marker genes after treatment, including *BCL2L1*, *PIK3CB*, and *STAT2* (Fig. 1H). The pre-treatment ancestors of the resistant clones largely lacked distinct transcriptional programs (Fig. 1F, right), suggesting that cancer cell heterogeneity may stem from stochastic and transient fluctuations in survival genes and pathways.

### *In vivo* model reveals clonal evolution and resistance emergence during MRD

**Fig. 2.**
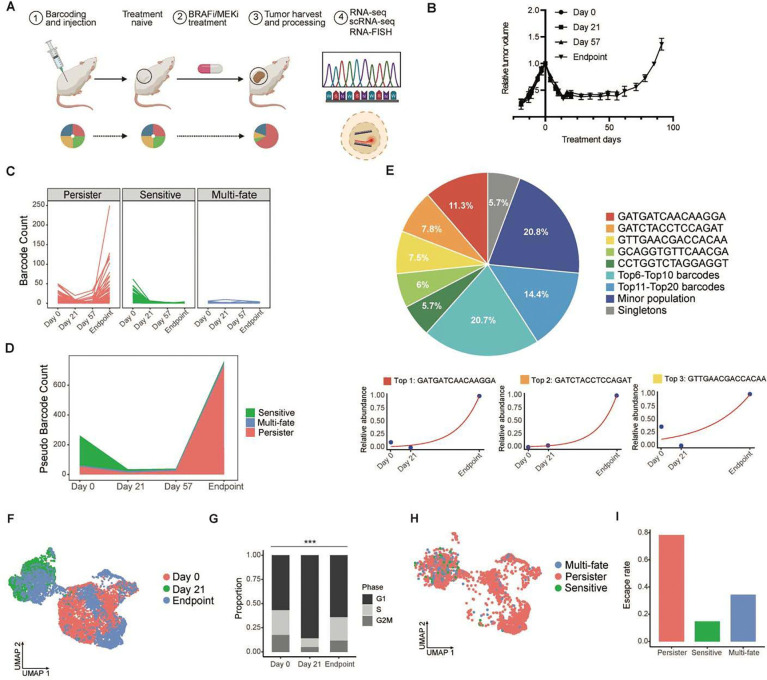
Clonal dynamics reveal the emergence of adaptive resistance. **a**, Experimental outline^13^: (1) Barcoded WM4237–1 cells were injected subcutaneously into NSG mice. (2) BRAFi/MEKi treatment and development of resistance. (3) Tumors were excised and processed at multiple time points. (4) Transcribed barcodes were sequenced and visualized *in situ*. Pie charts at the bottom illustrate the changes in barcode distribution during treatment. **b**, Growth curve of barcoded WM4237–1 tumors before and after BRAFi/MEKi treatment. Tumor collection time points: Day 0 (pre-treatment), n = 3; Day 21 (early MRD), n = 3; Day 57 (pre-recurrence), n = 3; and endpoint (resistant), n = 6. **c**, Normalized abundance of individual barcodes revealed distinct clonal trajectories: (1) persister subpopulations that initially regressed but later expanded under treatment; (2) sensitive subpopulations eliminated by BRAFi/MEKi; and (3) multi-fate subpopulations that fluctuated in abundance without dominating the resistant tumors. **d,** Stacked plot illustrating clonal dynamics driving tumor growth during BRAFi/MEKi treatment. **e**, Top panel: Pie chart illustrating the hierarchical clonal composition of endpoint tumors from scRNA-seq data. The 15-bp 3′ end sequences of the five most dominant barcodes are shown on the right, while the proportions of barcodes ranked 6–10, 11–20, minor populations, and singletons were grouped respectively; Bottom panel: normalized abundance of top-ranked barcodes (ranks 1–3) across prolonged treatment. **f**, UMAP to visualize single-cell transcriptome clusters. Day 0 (pre-treatment), Day 21 (early MRD), and Endpoint (resistant). **g**, Proportion of cells in each cell cycle phase inferred from scRNA-seq. ***P < 0.001; two-tailed Fisher's exact test. **h**, UMAP visualization of subpopulations from the endpoint tumor including persister (red), sensitive (green), and multi-fate (blue) cells. **i**, Proportions of each endpoint subpopulation escaping the non-proliferative MRD state and re-entering a proliferative state resembling the pre-treatment tumor.

We leveraged the patient-derived xenograft (PDX) model WM4237–1^7^ (Table S3), which recapitulates the patient’s response to BRAFi/MEKi therapy, initially showing strong sensitivity until relapse. Replicates of MeRLin-barcoded WM4237–1 cells were injected subcutaneously into immunodeficient NSG (NOD-*scid* IL2Rgamma^null^) mice and formed tumors (Fig. 2A; Methods). The barcoded tumors initially responded to BRAFi/MEKi treatment but relapsed after approximately three months (Fig. 2B).

We conducted longitudinal studies on WM4237–1 tumors harvested at key time points: pre-treatment (day 0), early MRD (day 21), late MRD or pre-recurrence (day 57), and the resistant endpoint. Bulk RNA-seq was performed on the original control, three replicates from day 0, day 21, and day 57, along with six replicates from the endpoint (Fig. S3A). By retrieving barcode reads from bulk RNA-seq data (Methods and Table S4), we determined that 37% of transplanted clones were successfully engrafted into day 0 tumors, while only 12% of clones from pre-treatment tumors persisted through prolonged BRAFi/MEKi treatment, as detected in the resistant tumors (Fig. S3B).

Shannon index showed a decrease in barcode diversity at the early MRD stage compared to pre-treatment (Fig. S3C). Barcode diversity declined further by pre-recurrence, reaching levels similar to those in resistant tumors (Fig. S3C), indicating a reduction in clonal heterogeneity during MRD as resistance develops. Only six of approximately 200 barcodes were shared across all six resistant tumor replicates (Fig. S3D), suggesting that tumor recurrence is unlikely to be driven by pre-existing resistant clones present in pre-treatment tumors^6^.

We quantified the barcoded subpopulations by normalizing their proportions to the corresponding tumor volumes and global transcriptional activity across time points (Methods and Fig. S3F). Clonal dynamics were classified into three categories (Fig. 2C): (1) persister subpopulations, which initially responded to treatment but persisted and expanded during the MRD stage, ultimately dominating tumor recurrence; (2) sensitive subpopulations, which were eliminated by BRAFi/MEKi; and (3) multi-fate subpopulations, which fluctuated or persisted without becoming dominant in resistance. The stacked plot illustrates the clonal dynamics of all three subpopulations, showing that dominant resistant clones primarily arose from minor subpopulations in pre-treatment tumors, whereas sensitive subpopulations were eliminated by BRAFi/MEKi (Fig. 2D).

Among the six shared barcodes, three subpopulations belonged to persister cells (Fig. S3E), while the other three were classified as multi-fate subpopulations. These multi-fate cells were inherently resistant to BRAFi/MEKi, maintaining a relatively stable but slow growth rate during treatment without giving rise to dominant resistant clones. Our analysis distinguished adaptive mechanisms from pre-existing resistance, highlighting the former as the primary driver of tumor recurrence.

We applied RNA-MuTect to identify new somatic mutations in exons from the bulk RNA-seq data of the six resistant endpoint tumor replicates (Methods; Table S5). Although each tumor relapsed within approximately three months and acquired 200 to 300 genetic alterations in the coding regions, no shared mutations were detected (Fig. S3G).

### Single-cell analysis reveals transcriptional trajectories of persister subpopulations

We performed scRNA-seq on samples from treatment day 0, day 21, and the endpoint, pooling three replicate tumors per time point to characterize transcriptomic changes over time (Methods). Through scRNA-seq data, Shannon index similarly demonstrated a decrease in barcode diversity on day 21 compared to the pre-treatment tumor, and a further decline in the recurrent tumor (Fig. S4A). We detected 261 unique barcodes in the recurrent tumor, compared to 1,127 unique barcodes in the pre-treatment tumor, indicating that only 23% of the initial subpopulations survived prolonged BRAFi/MEKi therapy (Table S4). Resistant clones at the endpoint displayed a hierarchical distribution, with the most dominant clone comprising 11.3% of the tumor and the top five persister lineages collectively accounting for 40% of the recurrent tumor (Fig. 2E). The three top-ranked persister subpopulations underwent exponential growth during prolonged BRAFi/MEKi treatment (Fig. 2E; Methods). We traced the clonal dynamics of individual lineage barcodes across different time points and classified them into persister, sensitive, and multi-fate subpopulations (Fig. S4B).

Single cell transcriptomics revealed distinct transcriptional states between early MRD (day 21) and pre-treatment (day 0) tumors, whereas cells from the resistant (endpoint) tumors were located across both transcriptional states (Fig. 2F). Cell cycle phase inferred from scRNA-seq data demonstrated a higher proportion of cycling cells in G2/M and S phases in the populations of day 0 and endpoint tumors, while day 21 cells predominantly exhibited G1 cell cycle arrest (Fig. 2G; Methods). Uniform manifold approximation and projection (UMAP) of endpoint tumor subpopulations with distinct clonal fates revealed that cells classified as persisters were uniquely capable of escaping the non-proliferative MRD state and re-entering a proliferative state resembling the pre-treatment tumor (Fig. 2H). In contrast, the sensitive and multi-fate subpopulations remained largely confined to the non-proliferative MRD state (Fig. 2I).

### Integrated lineage-transcriptome analysis uncovers diverse persister programs

**Fig. 3.**
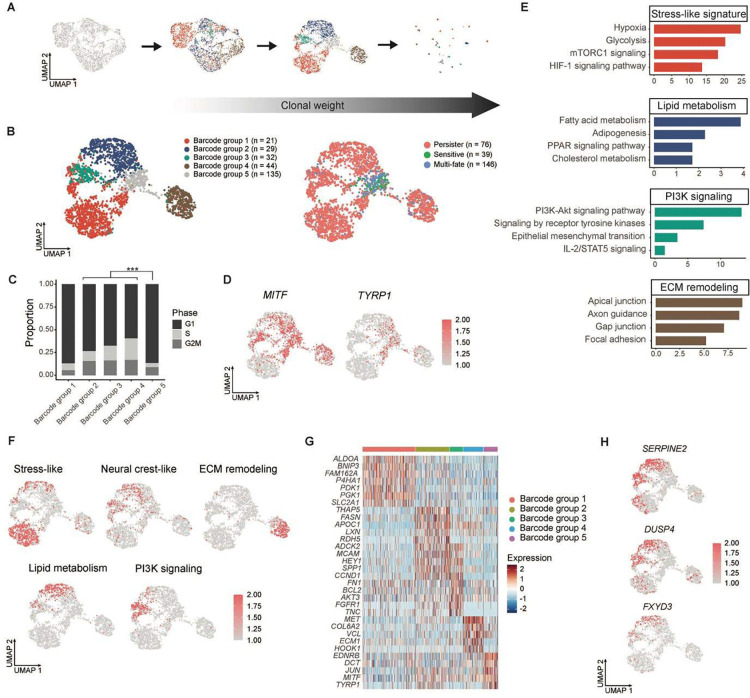
DTP cells acquire resistance via distinct transcriptional programs. **a,** ClonoCluster was used to incorporate clonal barcode information (colored) into the transcriptome (grey). UMAPs show the effect of increasing Warp Factor values (0, 6, and 10) on the structure of endpoint tumor scRNA-seq data (Methods). **b,** UMAP visualizations of hybrid clusters (left) integrating clonal and transcriptomic information and their clonal fate (right). Barcode groups 1–4 (red, blue, green, brown in the left panel) represent persister subpopulations (red in the right panel), whereas barcode group 5 (gray in the left panel) corresponds to sensitive (green in the right) and multi-fate (blue in the right) subpopulations. n, unique barcode number in each barcode group or clonal fate. **c,** Proportion of cells in each cell cycle phase inferred from scRNA-seq. *** P < 0.001; two-tailed Fisher's exact test. **d,** Cells on the UMAP recolored by the expression of the melanoma differentiation markers *MITF* and *TYRP1*. **e,** Functional enrichment terms of barcode groups 1–4 identified in **b**. P-values were determined using two-tailed Fisher’s exact tests (Table S8). **f,** The AUCell scores (color scale) of the top functionally enriched programs per state projected on UMAP (Table S7). **g,** Discriminative marker genes (n = 5–7) for each barcode group (Table S6). Fold change ≥ 2, cell percentage ≥ 50%, and FDR < 0.05. **h**, UMAPs showing *SERPINE2*, *DUSP4*, and *FXYD3* expression within the barcoded WM4237–1 persister subpopulations.

We applied ClonoCluster, a clustering algorithm that integrates clone and transcriptome information into hybrid clusters (Fig. 3A; Methods). The resulting clusters were used to reduce cell type entropy and identify biologically relevant markers^14^ (Fig. S5A–S5C). ClonoCluster improved the separation of hybrid clusters comprising clonally and transcriptionally similar subpopulations through different stages of BRAFi/MEKi treatment. In recurrent tumors, we identified five barcode groups using hybrid clustering (Fig. 3B, left). Barcode groups 1 to 4 corresponded to persister subpopulations, and barcode group 5 consisted of sensitive and multi-fate subpopulations (Fig. 3B, right and Fig. S5D).

Examining cell cycle phases, we found that in recurrent tumors, persister subpopulations from groups 1 to 4 showed an overall higher fraction of cycling cells in the G2/M and S phases, whereas sensitive and multi-fate subpopulations in group 5 predominantly underwent G1 cell cycle arrest (Fig. 3C; Methods). We detected a wide variability in the expression levels of *MITF*, a key regulator of melanoma differentiation^15^, across all subpopulations in the recurrent tumor (Fig. 3D, left). Persister cells from group 1 exhibited the lowest *MITF* expression, indicating that these cells were the most dedifferentiated among all subpopulations. We also found that *TYRP1*, a differentiation marker and *MITF* target gene, was enriched in the sensitive and multi-fate subpopulations (Fig. 3D, right).

To characterize the cellular expression programs underlying persister subpopulations, we identified differentially expressed genes in each group (Methods; Table S6). EnrichR pathway analysis was used to functionally annotate each cluster (Methods; Table S7), revealing that group 1 was enriched for stress-like signatures^16^, groups 2 and 3 shared neural crest stem-like (NC-like) features^16^, and group 4 was associated with extracellular matrix (ECM) remodeling states (Fig. 3E and Table S8). The activities of these expression programs were measured using AUCell and projected onto UMAP space (Fig. 3F; Methods). The transcriptional states of groups 2 and 3 were associated with lipid metabolism and PI3K signaling, respectively^17^ (Fig. 3E and 3F).

### Persister states engage diverse programs to drive resistance

Persister cells from barcode group 1 exhibited a stress-like state, characterized by the expression of markers, such as *BNIP3* and *PDK1*^18^ (Fig. 3G and Fig. S6B; Table S6). Upregulation of *PDK1*, *SLC2A1*, and *ALDOA* contributes to the Warburg effect by promoting a metabolic shift toward glycolysis over oxidative phosphorylation^18^. In melanoma and other cancers, *BNIP3* and *P4HA1* are induced under hypoxic conditions, promoting tumor growth and progression^18^. A recent study showed that a subset of melanoma cells rapidly escapes BRAF inhibition by relying on *ATF4*-mediated stress signaling to sustain periodic cycling under drug pressure^19^.

Cells from barcode groups 2 and 3 shared a neural crest stem-like signature^16^ (Fig. 3F), marked by the expression of dedifferentiation markers *MCAM* (CD146), *HEY1*, and *SPP1* (osteopontin) (Fig. 3G and Fig. S6C; Table S6), which are associated with adverse clinical outcomes in melanoma^20^, suppression of neuronal differentiation^21^, and maintenance of a stem-like proliferative state^22^. Although they largely overlapped, the two NC-like subpopulations presented distinct molecular features. Barcode group 2 uniquely expressed lipid metabolism genes, such as *FASN* and *APOE* (Fig. 3G and Fig. S6D; Table S6), which contribute to melanoma resistance by promoting MAPK inhibitor resistance through reducing lipid poly-unsaturation and protecting *MITF*^low/*AXL*ĥigh persister cells from ferroptosis, respectively^23,24^. Barcode group 3 cells were enriched for PI3K signaling markers, such as *AKT3* and the receptor tyrosine kinase *FGFR1* (Fig. 3G and Fig. S6E; Table S6). *AKT3* promotes resistance to apoptosis in BRAF-targeted melanoma cells, and FGF/FGFR signaling supports melanoma survival and therapy resistance^25,26^.

Persister cells from barcode group 4 uniquely expressed genes involved in extracellular matrix (ECM) remodeling, including *ECM1*, *VCL* (vinculin), and *MET* (c-MET) (Fig. 3G and Fig. S6F; Table S6). *ECM1* supports cancer stem cell maintenance by enhancing β-catenin signaling, thereby promoting epithelial-mesenchymal transition (EMT) and drug resistance^27^. Vinculin facilitates tension-dependent ECM remodeling via mechanosignaling and c-MET drives cancer progression regulated by ECM components^28^.

Furthermore, transcriptomic profiling enabled by MeRLin identified 200 novel marker genes with significantly increased expression in persister cells compared to sensitive and multi-fate subpopulations (Table S6, Barcode groups 1–4). Persister subpopulations exhibited increased expression of *SERPINE2*, *DUSP4* and *DUSP6* (Fig. 3H and Fig. S6G). Consistent with these findings, our lab has previously identified *SERPINE2* as a marker of label-retaining cells critical for drug resistance^29^. Notably, we identified differentially expressed cell surface markers including *MCAM* (CD146), *CSPG4*, and *ITGA6/ITGA7*, which are well-established melanoma resistance markers (Fig. S6C and S6G). We also identified *FXYD3* as a potential cell surface biomarker of resistance (Fig. 3H), which was significantly upregulated in persister subpopulations.

### Transcriptional and epigenetic regulation underlies persister states

**Fig. 4.**
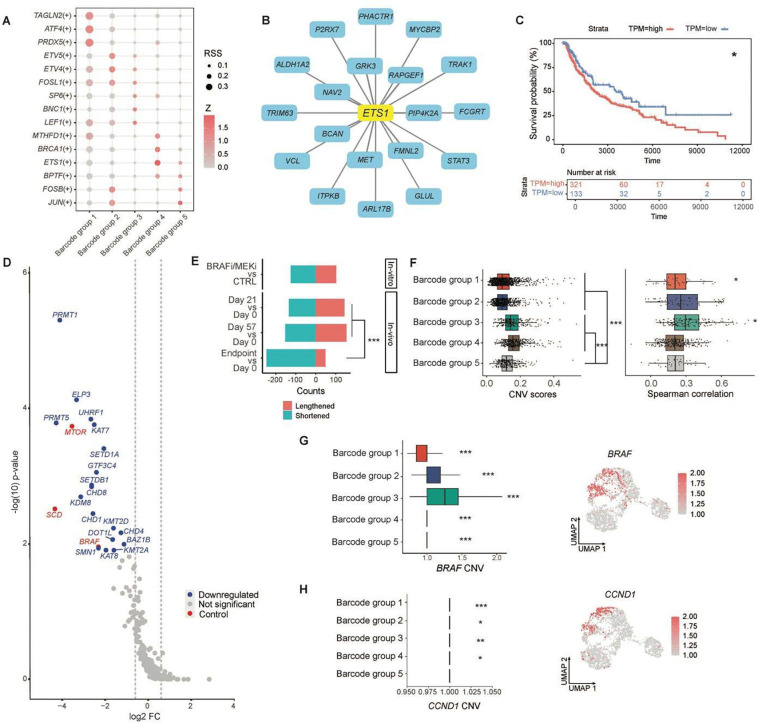
Regulatory mechanisms underlying adaptive resistance. **a,** SCENIC analysis identifying key regulators of each barcode group. **b,** Regulon activity of *ETS1* on its target genes in the ECM remodeling state. **c,** Survival analysis of TCGA data revealed that high *ETS1* expression was associated with worse patient outcomes (P = 0.018). **d**, Volcano plots showing depleted sgRNAs from the CRISPR screen, highlighting the top negatively selected genes. Log_2_FC (fold change) < − 1, and 18 genes were identified with FDR < 0.15. **e**, Alternative polyadenylation (APA) analysis showing 3′UTR lengthening and shortening events across *in vitro* treatment and *in vivo* time points, compared to control cells and pre-treatment tumors. ***P < 0.001; two-tailed Fisher's exact test. **f**, Copy number variation (CNV) scores for each barcode group (left). ***P < 0.001; two-tailed t-test. Spearman's rank correlation coefficients (rs) between CNV scores and gene expression in each barcode group (right). rs_1_ = 0.21, P_1_ = 0.047; rs_3_ = 0.28, P_3_ = 0.012 (median rs, subscripts indicate barcode groups). **g**, Copy number variation (CNV) scores of *BRAF* in each barcode group (left). Spearman's rank correlation coefficients between CNV and *BRAF* expression: rs_1_ = 0.44, rs_2_ = 0.56, rs_3_ = 0.65, rs_4_ = 0.23, rs_5_ = 0.29, (subscripts indicate barcode groups), ***P < 0.001. UMAP (right) shows *BRAF* expression. **h**, Copy number variation of *CCND1* in each barcode group (left). Spearman's rank correlation coefficients between gene expression and CNV of *CCND1* in each barcode group: rs_1_ = 0.23, rs_2_ = 0.1, rs_3_ = 0.17, rs_4_ = 0.13 (subscripts indicate barcode groups). *P < 0.05, **P < 0.01, ***P < 0.001. UMAP (right) shows *CCND* expression.

SCENIC analysis revealed clear distinctions among stress-like, lipid metabolism, PI3K signaling, ECM remodeling, and melanocytic states (Methods; Fig. S7A). Specifically, *ATF4* was identified as the key regulator of the stress-like state^19^, *ETV5* for lipid metabolism^30^, *LEF1* for PI3K signaling^31^, and *JUN* for melanocytic state^32^ (Fig. 4A). Unsupervised analysis predicted *ETS1* as the transcription factor driving ECM remodeling in melanoma^33^. A set of *ETS1* target genes were identified, including *VCL* and *MET* (Fig. 4B; Methods). Notably, five of the top ten genes most highly expressed in group 4 were *ETS1* targets, supporting a role for *ETS1*-driven ECM remodeling in resistance. Elevated *ETS1* expression was also associated with poorer patient survival in TCGA dataset (Fig. 4C).

Our findings suggested that adaptive mechanisms are the primary drivers of cancer resistance. In particular, epigenetic reprogramming in DTP cells enables adaptive resistance by introducing heritable changes that promote cellular plasticity^3^. To systematically identify the key drivers of melanoma plasticity and resistance, we performed a custom CRISPR screen using a restricted library targeting 235 epigenetic regulators that were expressed in melanoma and were considered potentially druggable genes (PDGs)^34,35^ (Methods; Table S10; Fig. S7B). Depletion of the top hit-targeting sgRNAs identified 18 genes whose loss sensitized BRAF V600E-mutant WND238 cells to BRAFi/MEKi (Fig. 4D and Table S3). These include key histone methyltransferases, such as *PRMT1*, *PRMT5*, *DOT1L*, and *SETDB1*^36–38^. Notably, *PRMT1* and *DOT1L* were highly expressed in persister cells across barcode groups 1–4 (Fig. S7C), and survival analysis of TCGA data showed that elevated expression of *PRMT1* and *DOT1L* correlated with poorer patient outcomes (Fig. S7D). Together, these findings identified *PRMT1* and *DOT1L* as potential therapeutic targets for overcoming epigenetically driven drug resistance in melanoma^36^.

### Post-transcriptional regulation and copy number variation contribute to adaptive resistance

Next, we investigated the post-transcriptional mechanisms underlying adaptive resistance. scRNA-seq data were used for mining alternative polyadenylation (APA) isoform expression, which is a widespread regulatory mechanism that generates distinct 3′ transcript ends and is closely linked to cell identity, proliferation, and differentiation^39^ (Methods). We observed both 3′UTR lengthening and shortening events during the MRD stage (days 21 and 57) compared to pre-treatment tumors, as well as in BRAFi/MEKi-treated melanoma cells compared to untreated controls *in vitro* (Fig. 4E). With prolonged treatment, global transcript isoforms in recurrent tumors were significantly shortened. Notably, 3'UTR shortening is known to be a key regulatory mechanism in proto-oncogene activation, enabling proliferative genes to bypass miRNA-mediated repression^40^.

The copy number variation (CNV) content of each barcode group was ascertained from scRNA-seq data using inferCNV (Methods), indicating higher somatic copy number alterations in barcode groups 3 and 4 (Fig. 4F, left). In group 3, higher gene expression was significantly correlated with CNV, whereas no such correlation was observed in group 4 (Fig. 4F, right). Notably, increased *BRAF* expression was strongly associated with its copy number amplification in groups 2 and 3 (Fig. 4G). In contrast, the elevated expression of *CCND1* (cyclin D1), a key cell cycle regulator, showed only a weak correlation with CNV in these groups (Fig. 4H).

### Longitudinal clonal tracking reveals early emergence and reprogramming of persisters

**Fig. 5.**
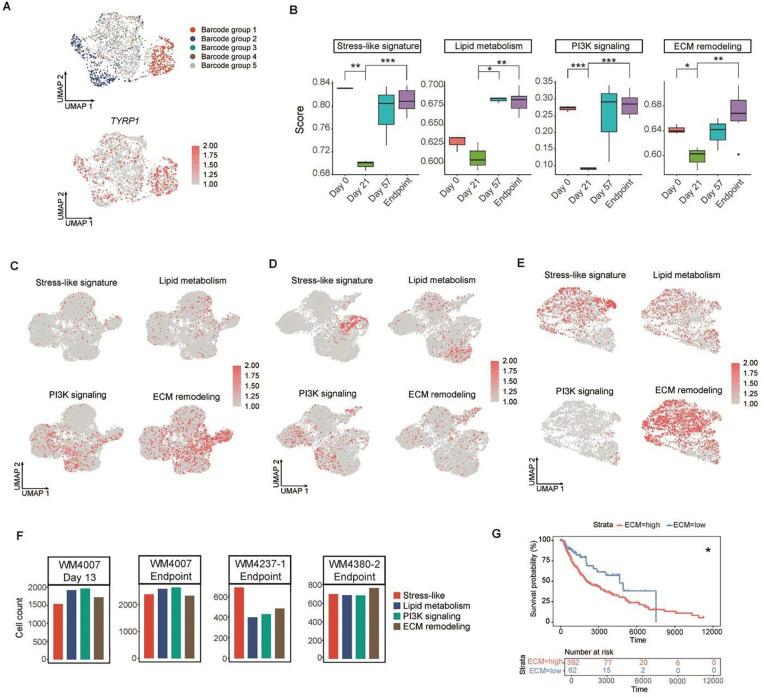
Persister states occur through treatment and across models. **a**, ClonoCluster (top panel) applied to scRNA-seq data from day 21 barcoded WM4237–1 tumors. UMAPs show barcode groups 1–5 (red, blue, green, brown, gray) from endpoint tumors; UMAP (bottom panel) illustratesing upregulated *TYRP1* expression in barcode group 1 in the day 21 barcoded WM4237–1 tumor. **b**, Pathway enrichment scores derived from bulk RNA-seq of barcoded WM4237–1 tumors across BRAFi/MEKi treatment. *P < 0.05, **P < 0.01, ***P < 0.001; two-tailed t-test. **c**, UMAPs showing persister states from non-barcoded WM4007 scRNA-seq at endpoint and day 13 MRD stage (**d**). **e**, UMAPs illustrating persister signatures from non-barcoded WM4380–2 scRNA-seq data at the endpoint. **f**, Proportions of persister states across BRAF V600E-mutant PDX models WM4007 (day 13 and endpoint), WM4237–1, and WM4380–2. **g**, Survival analysis of TCGA data revealed that high expression of the ECM remodeling signature was associated with worse patient outcomes (P = 0.011).

Persister barcode groups identified from endpoint tumors of WM4237–1 were longitudinally tracked across multiple time points during BRAFi/MEKi treatment (Methods). Using ClonoCluster, which integrates clonal information with scRNA-seq data, we identified persister barcode groups 1 and 2 at the early MRD stage (day 21) (Fig. 5A), whereas groups 3 and 4 were scarcely detected. No barcode groups were distinguishable at the pre-treatment time point (day 0) (Fig. S8A). At day 21, among the two early-emerging persister subpopulations, barcode group 1 exhibited few differentially expressed genes, including upregulation of *TYRP1* (Fig. 5A), suggesting a more differentiated state^8^. In contrast, at the endpoint, barcode group 1 displayed the most dedifferentiated phenotype, characterized by the lowest *MITF* activity (Fig. 3D). These findings highlighted a dramatic phenotypic transition toward stress-like persister cells in barcode group 1 as they acquire adaptive resistance during progression from early MRD to tumor recurrence.

From the bulk RNA-seq data, we also profiled the gene expression signatures across multiple time points, including day 57, during BRAFi/MEKi treatment in the WM4237–1 model (Fig. 5B and S8B; Methods). Notably, these adaptive programs were significantly suppressed at the early MRD stage (day 21), when tumors were responding to therapy, compared to the late MRD stage (day 57) and tumor recurrence. Although the overall tumor volumes remained relatively stable throughout the MRD stage, persister cells exhibited substantial phenotypic plasticity through distinct transcriptional reprogramming.

### Persister programs are conserved across PDX models

Additionally, we validated these gene expression signatures in two independent non-barcoded scRNA-seq datasets from BRAF V600E-mutant PDX models (Methods; Table S3). The first was the BRAFi/MEKi-sensitive model WM4007, which showed an initial response to BRAFi/MEKi therapy, but relapsed after approximately seven months (Fig. S8C). Longitudinal tumors were similarly collected at four time points: pre-treatment (day 0), MRD (days 13 and 20), and the resistant endpoint. scRNA-seq analysis revealed that the recurrent tumors also exhibited stress-like, lipid metabolism, PI3K signaling, and ECM remodeling states (Fig. 5C). Notably, these signatures were already detectable at the early MRD stage on days 13 and 20 (Fig. 5D and Fig. S8E). The second model was the BRAFi/MEKi-resistant WM4380–2 (Fig. S8D), derived from a stage IV melanoma patient previously treated with BRAFi^41^ (Table S3). As expected, all four transcriptional states were present in this model, with the resistant tumor exhibiting a particularly strong ECM remodeling state (Fig. 5E). In comparison, the recurrent WM4237–1 tumor predominantly displayed the stress-like state, whereas WM4007 tumors showed sustained activation of PI3K signaling throughout prolonged treatment (Fig. 5F). Notably, elevated expression of the ECM remodeling signature was associated with poorer patient outcomes in TCGA dataset. (Fig. 5G).

### Spatial mapping of adaptive programs and signaling networks in recurrent melanoma

**Fig. 6.**
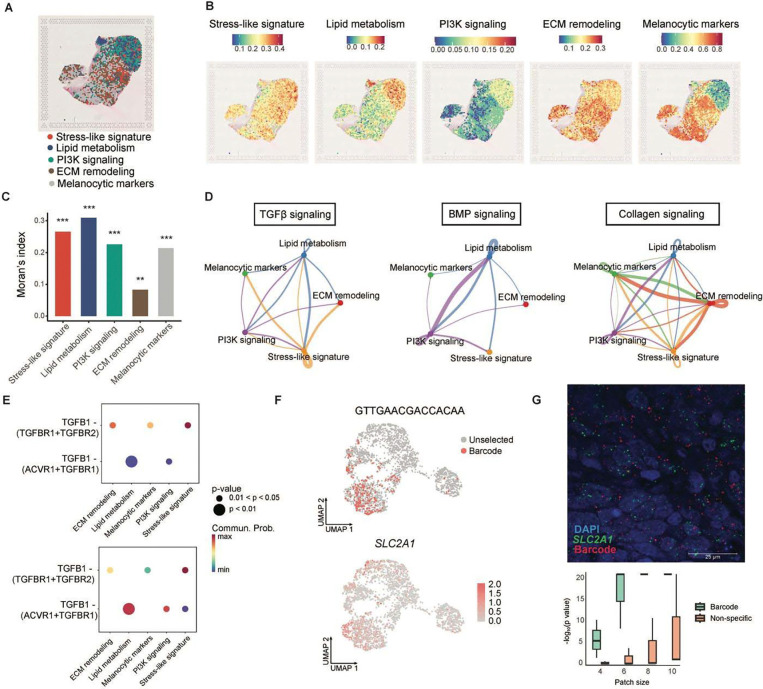
Spatial profiling of diverse drug-tolerant persister states. **a**, Spatial transcriptomics showing persister states overlaid on H&E-stained sections of a recurrent WM4237–1 PDX tumor. Stress-like (red), lipid metabolism (blue), PI3K signaling (green), ECM remodeling (brown), and melanocytic (gray) states. **b**, Distinct spatial distribution of each persister state with quantification scale. **c**, Moran's indices showing spatial autocorrelation for each persister state. Stress-like (red), I = 0.27; lipid metabolism (blue), I = 0.31; PI3K signaling (green), I = 0.23; ECM remodeling (brown), I = 0.083; and melanocytic (grey), I = 0.21. **P < 0.01, ***P < 0.001. **d**, Chord diagrams reveal the strength of cell-cell communication among persister states. Stress-like (yellow) and lipid metabolism (blue) states emitted TGFβ signals (left); lipid metabolism (blue) and PI3K signaling (purple) states exhibited autocrine and paracrine BMP signaling (middle); and ECM remodeling state (red) sent collagen signals (right). **e**, Specific ligand-receptor pairs involved in TGFβ signaling in **d** (left). Outgoing TGFβ signaling from the stress-like state (top) and lipid metabolism state (bottom). **f**, UMAPs illustrating the expression of the dominant barcode GTTGAACGACCACAA-3 and the stress-like marker *SLC2A1*. **g**, RNA-FISH (top panel) of a recurrent WM4237–1 barcoded tumor revealed co-localization of a resistant clone (barcode: GTTGAACGACCACAA-3) and the stress-like marker *SLC2A1*. Statistical analysis (bottom panel) showed significant co-localization between *SLC2A1* and the resistant clone across all patch sizes (Fisher’s exact test, P < 0.014), compared to co-localization with a non-specific probe set (patch size of 10 × 10, Fisher's exact test, P = 0.089). The analysis was based on three ROIs (regions of interest) each.

To characterize the spatial organization of the adaptive programs, we annotated stress-like, lipid metabolism, PI3K signaling, and ECM remodeling states using spatial transcriptomics (Methods). A published PDX dataset of WM4237–1 following prolonged BRAFi/MEKi treatment revealed the presence of these distinct transcriptional states in recurrent tumors^42^ (Fig. 6A and Fig. S9B). Stress-like signatures showed a scattered distribution, whereas the ECM remodeling state formed a mosaic pattern interspersed with melanocytic cells (Fig. 6B). Notably, lipid metabolism and PI3K signaling signatures associated with the NC-like state were colocalized and enriched near the tumor boundary (Fig. 6B). Moran’s index revealed that the ECM remodeling state exhibited the weakest spatial autocorrelation among the signatures, indicating the lowest degree of spatial dependence (Fig. 6C; Methods).

We used CellChat to quantitatively infer intercellular communication networks among persister states (Methods). The outgoing signaling of TGFβ and Midkine (*MK*) was primarily observed in stress-like cells (Fig. 6D and Fig. S10A). Previous studies have shown that autocrine TGFβ production by melanoma cells can activate local fibroblasts into myofibroblasts, promoting fibronectin deposition and establishing a resistant tumor niche^43^. Cell-cell communication between lipid metabolism and PI3K signaling states included BMP (bone morphogenetic protein), ANGPTL (angiopoietin-like protein), CSPG4 (chondroitin sulfate proteoglycan 4), and MPZ (myelin protein zero, P0), all of which are associated with neural differentiation^44^ (Fig. 6D and Fig. S10B). Notably, *MPZ* was upregulated among the differentially expressed genes in lipid metabolism associated barcode group 2 (Table S6). Collagen and Claudin (CLDN) signaling was strongly upregulated in the ECM remodeling state, contributing to the switch to a differentiated and proliferative phenotype^45^ (Fig. 6D and Fig. S10C). We characterized the specific ligand-receptor pairs involved in TGFβ1 signaling and found that the outgoing signaling pattern of stress-like cells primarily involved TGFβR1 (ALK5) and TGFβR2, whereas the lipid metabolism state showed outgoing signaling through either TGFβR2 or ACVR1 in combination with TGFβR1 (Fig. 6E).

### MeRLin enables spatial clonal profiling and validation of persister states

Cellular barcoding technologies are limited by barcode measurement constraints, as sequencing retains sequence information but loses spatial context, whereas imaging preserves spatial organization but has lower sensitivity to barcode sequences. Our MeRLin system, consisting of 265 bp semi-random sequences, enabled simultaneous clonal profiling and gene expression *in situ*. Guided by bulk or single-cell RNA-seq data, we were able to recover the full-length barcode sequence of a selected subpopulation (Methods). We designed RNA-FISH probes targeting the specific barcode and used them to visualize the corresponding cells. Using RNAscope fluorescent multiplex assay, we spatially mapped a dominant persister subpopulation (Fig. 2E, top 3 barcode, 7.5% of recurrent tumor) and quantified the co-occurrence of the stress-like marker *SLC2A1* (Fig. 6G). Statistical analysis revealed significant co-localization between the dominant barcode and *SLC2A1* expression in the recurrent tumor (Fig. 6G). Consistent with scRNA-seq results, *SLC2A1* was upregulated in the selected persister subpopulation (Fig. 6F), supporting its association with the stress-like transcriptional program in melanoma resistance (Fig. 3F). Importantly, in combination with our computational strategy to identify representative markers of persister subpopulations, we demonstrated that barcoded RNA-FISH enabled *in situ* clonal profiling and single-cell transcript quantification.

## Discussion

Under sustained targeted therapy, cancer cells do not merely endure drug pressure, but actively adapt, reprogram, and evolve^3^. Drug-tolerant persister (DTP) cells emerge as key survivors, reshaping signaling pathways and cellular identities to evade therapeutic inhibition^16^. However, elucidating the dynamic reprogramming of these cells over time remains a major challenge.

To illuminate the evolving landscape of resistance, we developed MeRLin, a lineage tracing platform that integrates cellular barcoding, single-cell transcriptomics, and RNA-FISH to simultaneously track clonal evolution, transcriptional plasticity, and spatial organization in melanoma under BRAF/MEK inhibition^6^. Using MeRLin in patient-derived xenograft models, we found that therapeutic resistance arises primarily through adaptive reprogramming, rather than the selection of pre-existing resistant clones. Dominant resistant clones originate from initially minor, transcriptionally homogeneous subpopulations that acquired resistance-associated features over time, giving rise to distinct persister states.

Integrated clonal and transcriptomic analysis revealed four major persister states characterized by stress-like, lipid metabolism, PI3K signaling, and extracellular matrix (ECM) remodeling programs that enable cells to circumvent therapeutic pressure^46^. The stress-like state, marked by *ATF4* activity and upregulation of *SLC2A1*, *PDK1*, and *BNIP3*, reflects metabolic adaptation and stress tolerance^16^. Lipid metabolism (*FASN* and *APOE*) and PI3K signaling (*AKT3* and *FGFR1*) states correspond to previously described neural crest-like cells associated with dedifferentiation and therapeutic resistance^16,17^. The ECM remodeling state, regulated by *ETS1*, featured the expression of *ECM1*, *VCL*, and *MET*. These states were dynamic and re-emerged before relapse, suggesting that they were early indicators of recurrence and actionable vulnerabilities.

Post-transcriptional regulation further shaped adaptive resistance. We observed transcriptome-wide 3′UTR lengthening during early minimal residual disease (MRD), followed by 3′UTR shortening in recurrent tumors, consistent with enhanced oncogenic activation^39^. PI3K-associated persisters also display increased somatic copy number alterations. Importantly, these four adaptive programs were conserved across the independent BRAF-mutant melanoma models. We also identified cell surface markers enriched in DTPs including *MCAM*, *CSPG4*, *ITGA6/7*, and *FXYD3*. Using a focused CRISPR screen for epigenetic regulators, we identified *PRMT1*, *DOT1L*, and *SETDB1* as functional dependencies of resistance. Together, these findings highlight the dynamics and plasticity of the DTP states and reveal their potential vulnerabilities.

Although the WM4237–1 model was used in both *in vivo* and *in vitro* settings, notable differences were observed. Global 3′UTR shortening seen in recurrent tumors was absent in BRAFi/MEKi-treated resistant clones *in vitro* (Fig. 4E), which instead upregulated melanocytic genes (Fig. S2A). In contrast, these genes were downregulated in the persister subpopulations *in vivo* (Fig. 3D and Fig. S6A). While *in vitro*-treated cells adopted a transcriptional state entirely distinct from untreated controls (Fig. 1B), endpoint tumors retained partial similarity to pre-treatment tumors (Fig. 2F). Early MRD-stage (day 21) tumors exhibited a distinct state compared to pre-treatment tumors, indicating a dynamic transition toward the resistant phenotype observed in endpoint tumors (Fig. 2F).

Spatial transcriptomics showed the architectural organization of persister states. Lipid metabolism and PI3K signaling states co-localized near the tumor boundary, while ECM-remodeling cells showed the lowest spatial dependence. A complex signaling network, where stress-like cells secrete TGFβ and Midkine, and ECM-remodeling cells engage Collagen and Claudin pathways, establishes a niche that sustains resistance through autocrine and paracrine interactions. Additionally, MeRLin-enabled barcoded RNA-FISH allowed *in situ* visualization of clonal identity and quantification of gene expression. We observed spatial co-localization of the stress-like marker *SLC2A1* with a dominant resistant clone, linking the molecular phenotype to clonal origin and spatial context.

In summary, our study joins a growing body of literature on multidimensional characterization of tumor cell heterogeneity and the interplay between selection and phenotypic plasticity in cancer progression and treatment^4,18,47^. By linking clonal fate with transcriptional and spatial identity at single-cell resolution, MeRLin revealed the trajectories of melanoma resistance and uncovered vulnerabilities to disrupt persister evolution before stable resistance is established^1^.

## Methods

### Cloning of the MeRLin library

We generated the pCDH-EF1a-eFFly-mNeptune2.5 lentiviral vector by replacing mCherry in pCDH-EF1a-eFFly-mCherry^48^ (Addgene #104833, a gift from Irmela Jeremias) with mNeptune2.5^49^ (Addgene #51310, a gift from Michael Lin) using NsiI and SalI restriction sites (NEB) (Table S1). To optimize the simultaneous sequencing of barcodes by scRNA-seq, we mutated the original 3′ LTR KpnI site (C-to-T at position 3931) and introduced a new KpnI site (6-nt insertion at position 3961) using the Q5 Site-Directed Mutagenesis Kit (NEB E0552). Two PAGE-purified Ultramer oligonucleotides (IDT) of barcode inserts containing semi-random repeat patterns and homologous flanks (Table S1) were cloned into the KpnI-digested backbone by Gibson Assembly using NEBuilder HiFi DNA Assembly Master Mix (NEB E2621). The protocol for cloning barcodes was adapted from published methods^50^ and is available at (https://www.protocols.io/view/barcode-plasmid-library-cloning-5qpvon6yxl4o/v1). Plasmid sub-libraries were pooled before lentivirus production, yielding a final library diversity of 2.89 million barcodes (Table S2).

### Cell culture

Melanoma WM4237–1 and WND238 cell lines were established in-house, cultured in TU2% media (80% MCDB 153, 10% L-15, 2% FBS, 2.4 mM CaCl ), and passaged with 0.05% trypsin-EDTA. Lenti-X 293T cells (Clontech) were maintained in DMEM (10% FBS) and similarly passaged for lentivirus production.

### Lentivirus packaging and transduction

Lenti-X 293T cells were cultured to ~80% confluency in 10 cm dishes. For transfection, the media were replaced with 10 mL DMEM (10% FBS) containing 25 μM chloroquine diphosphate for 5 h. A mixture of 81.6 μL Transporter 5 (Polysciences) in 420 μL Opti-MEM (Thermo Fisher Scientific, 31985062) was combined with 8.6 μg psPAX2, 2.6 μg pMD2.G, and 9.2 μg barcode plasmids in 500 μL Opti-MEM, incubated for 20 min, and added dropwise. After 18 h, the medium was replaced with DMEM (5% FBS). Virus-containing supernatants were collected at 48 and 72 h, pooled, centrifuged (500 × g, 5 min), filtered (0.45 μm PES filter), and concentrated with Lenti Concentrator (Origene, TR30025) in 4:1 ratio, incubated overnight at 4 °C. Pellets were spun (3,000 × g, 35 min) and resuspended in 1× DPBS at 1/20th of the original volume. Aliquots were stored at −80 °C.

Dissociated melanoma cells (225,000 cells/mL) were transduced with freshly thawed lentiviral library and 8 μg/mL polybrene, plated in six-well plates, and spinfected at 600 × g for 30 min at 32 °C to achieve an MOI < 40%. After overnight incubation at 37 °C, media was replaced with 2 mL TU2% and cells were expanded in 10-cm dishes. The mNeptune2.5-positive cells were sorted by flow cytometry. Barcoded cells were expanded and split into replicates, with one replicate saved as the original control.

### *In vitro* drug treatment

Stock solutions of 10 mM dabrafenib (NSC 764134, NCI) and 100 μM trametinib (NSC 758246, NCI) were prepared in DMSO and diluted in culture medium to final concentrations of 0.3 μM and 30 nM, respectively. WM4237–1 cells were treated for one week with the media refreshed every two days. After treatment, the cells were trypsinized, washed, and incubated with dead cell removal antibody (Miltenyi Biotec, Dead Cell Removal Kit), according to the manufacturer’s instructions. After final centrifugation (250 × g, 5 min), the pellets were resuspended in 80 μL PBS, transferred to tubes on ice, and counted prior to bulk RNA-seq and scRNA-seq.

### Animal studies

All animal procedures were approved by the Institutional Animal Care and Use Committee (IACUC, #201546). Cells (5 × 10 in 100 μL RPMI:Matrigel (Corning), 1:1) were subcutaneously injected into the right flanks of 6–8-week-old male NSG mice. The mice were received from the in-house breeding facility and housed under pathogen-free conditions. Tumor volumes were monitored using calipers and calculated as (W^2^ × L)/2. Upon reaching ~500 mm^3^, the mice were treated with a rodent diet containing 150 mg/kg dabrafenib and 1.5 mg/kg trametinib (Bio-Serv, S7581). For barcoded WM4237–1, tumors were harvested and cut into chunks on Days 0, 21, 57, and 91 (endpoint) after euthanasia of mice for bulk RNA-seq and formalin-fixed paraffin-embedded (FFPE) samples.

### Single-cell sample preparation

For single-cell preparation, tumor chunks from three replicates were pooled and minced. Approximately 0.2–1 g of tissue was dissociated in 5 mL enzyme mix (Miltenyi Biotec, Tumor Dissociation Kit) and processed for 1 h using a gentleMACS Octo Dissociator (Miltenyi Biotec) with heating. After adding 8 mL serum-free media, single-cell suspensions were filtered through (70 μm nylon mesh), centrifuged (250 × g, 7 min), and RBC-lysed with 1 mL ACK Lysing Buffer (Quality Biological, 118–156-101), followed by dilution and centrifugation (250 × g, 5 min). Pellets were resuspended in BSA buffer and incubated with mouse antibody cocktail (Miltenyi Biotec, Mouse Cell Depletion Kit) for 15 min at 4 °C. Labeled cells were passed through LS columns to enrich human tumor cells, washed three times, and centrifuged (250 × g, 5 min). Dead cells were removed using the Dead Cell Removal Kit (Miltenyi Biotec) as described above. The final cell pellets were resuspended in 80 μL of PBS and transferred to tubes on ice for counting and scRNA-seq.

### Bulk RNA-seq

Libraries for whole transcriptome RNA sequencing were prepared using the Stranded Total RNAseq with Ribo-zero Plus kit (Illumina, San Diego, CA, USA) according to the manufacturer’s instructions, starting with an input of 700 ng of total RNA and 10 cycles of final PCR amplification. Library size was assessed using the 4200 Tapestation and High-Sensitivity DNA Assay (Agilent, Santa Clara, CA, USA). Concentration was determined using a Qubit Fluorometer 2.0 (Thermofisher, Waltham, MA). Next-generation sequencing with a paired-end 2 × 150 bp run length was performed on the NovaSeq X platform (Illumina, San Diego, CA). A minimum of 30 M reads per sample were acquired for each sample.

### Cloning of pooled gRNA libraries

We generated the lentiviral vector backbone CROPseq-Guide-EFS-eFFly-mNeptune2.5 by replacing SpCas9-P2A-EGFP in the CROPseq-Guide-EFS-SpCas9-P2A-EGFP plasmid (Addgene #99248, a gift from Alex Hewitt) with eFFly-mNeptune2.5 from pCDH-EF1a-eFFly-mNeptune2.5 using Gibson Assembly (Table S9). gRNAs were synthesized as 81-nt oPools Oligo Pools (IDT) with homology to the hU6 promoter and gRNA scaffold (Table S10). Pooled gRNA libraries were cloned following a published protocol^51^ and screened by PCR for 166 bp inserts (Table S9). The library coverage for epigenetic regulators was estimated at 568× and was subsequently used for lentiviral production.

### CRISPR screen and data analysis

To generate stable Cas9-GFP WND238 cells, we transduced the cells with Cas9-GFP lentivirus^52^ (pL-CRISPR.EFS.GFP, Addgene #57818, a gift from Benjamin Ebert) at an MOI < 0.7 and sorted for GFP-positive cells. Cells infected with the gRNA library (MOI < 0.4) were treated with 0.3 μM dabrafenib and 30 nM trametinib for one week to identify resistance and sensitization hits, following a published CRISPR screen protocol^51^. Genomic DNA with coverage above 1000× was extracted, quantified, and used for indel PCR amplification (Table S9). Purified PCR products were pooled and sequenced on a NextSeq 1000 (Illumina, San Diego, CA, USA) using a P1 100-cycle kit (dual indexing, single-end) with the following run parameters: 8 base pair × 8 base pair × 120 base pair.

For data analysis, we utilized the MAGeCK (Model-based Analysis of Genome-wide CRISPR-Cas9 Knockout) software suite to calculate negative and positive enrichment scores and the corresponding ranks^53^. These enrichment scores were derived using the Robust Rank Aggregation (RRA) method, which evaluates whether single-guide RNAs (sgRNAs) targeting a particular gene cluster are significantly at the top or bottom of the ranked list, more frequently than expected by chance. A lower RRA score indicates stronger enrichment or depletion of a gene, suggesting a more significant impact on the observed phenotype.

### Barcoded RNA-FISH

To validate the co-localization of *SLC2A1* with the second most dominant clone (barcode suffix “GTTGAACGACCACAA”), we used paired bulk RNA-seq and scRNA-seq to reconstruct the full-length 265 bp barcode. A custom 5ZZ anti-sense probe set Syn-T2v3 (ACD 1588531-C1) was designed by the ACD Probe Design Team and synthesized to target this barcode mRNA transcript for spatial mapping. Using the RNAscope^™^ Multiplex Fluorescent Reagent Kit v2 with TSA Vivid Dyes (ACD 323270), we visualized this persister subpopulation and quantified the expression of the stress-like gene *SLC2A1* (RNAscope^®^ Probe Hs-*SLC2A1*, ACD 423141-C2) in tissue sections from WM4237–1 endpoint tumors. A 5ZZ sense probe targeting the anti-sense barcode sequence (Syn-T2v3-sense, ACD 1587501-C1) served as the negative control for non-specific staining.

Probes were used with TSA Vivid Fluorophores 650 (ACD 323273) and TSA Vivid Fluorophore 570 (ACD 323272), respectively, and imaged using a Leica TCS SP8 X WLL confocal microscope (63× objective, 2× zoom; laser settings at 405 nm for DAPI, 550 nm and 649 nm for probes). Image analysis was performed by masking, patching, and counting colocalized red and green signals to determine the enrichment p-value. RNA-FISH images were segmented into 4×4, 6×6, 8×8, and 10×10 (non-overlapping) patches to quantify the co-localization of *SLC2A1* with barcode “GTTGAACGACCACAA” or non-specific control.

### Single-cell RNA-seq library preparation and read alignment

Single cell droplets were generated using the Chromium Next GEM single cell 3’ kit v3.1 (10x Genomics). cDNA synthesis and amplification, library preparation, and indexing were performed using the 10x Genomics Library Preparation kit (10x Genomics), according to the manufacturer’s instructions. The overall library size was determined using the Agilent Bioanalyzer 2100 and the high-sensitivity DNA assay, and libraries were quantitated using KAPA real-time PCR. Libraries were pooled and sequenced on the NovaSeq 6000 (Illumina, San Diego, CA, USA) using an S2 300 cycle kit (Illumina), paired end run with the following run parameters: 26 base pair × 8 base pair (dual index) × 280 base pair.

Reads alignment was performed using Cell Ranger v8.0.0 (10x Genomics). For *in vivo* PDX samples, raw sequencing reads were first aligned to a combined human-mouse reference genome (refdata-gex-GRCh38_and_GRCm39–2024-A) to identify and exclude mouse-derived reads. Cells with more than 20% of the reads mapped to the mouse genome were removed. For barcoded samples, the reads were aligned to a custom reference genome that included the barcode vector sequence integrated into the human genome (GRCh38–2024-A). All the other samples were aligned directly to the human genome (GRCh38–2024-A).

### Clonal barcode extraction

Clonal barcodes and their corresponding 10x cell barcodes (CB tags) were retrieved from the BAM files generated using Cell Ranger. Reads containing barcode sequences were aligned to a pseudo-chromosome that represented the barcode vector. We have developed a clonal barcode extraction tool to automate this process. Briefly, the extractor first scans for the known 3’ sequence of the vector backbone (CAGATCTTAGCCACTTTTTAAAAGAAAAGGGGG; Table S1) to locate the start of the semi-random clonal barcode sequence in each read. The extracted clonal barcode sequences were subsequently processed through a series of quality control and correction steps. For each high-confidence barcode, the first 15 bases and their corresponding CB tags were compiled into a structured data frame (details are available on GitHub).

### Computational analysis of scRNA-seq data

All scRNA-seq expression matrices were processed using the Seurat v5^54^. The barcode information was incorporated into the cell metadata of each Seurat object. Cells were filtered out if they had fewer than 2,000 detected genes, more than 50,000 unique molecular identifiers (UMIs), or over 20% of the reads mapped to mitochondrial genes. Doublets were identified and removed using DoubletFinder v2.0.4^55^. Cell cycle phase scores were calculated based on the canonical S and G2/M genes. After quality control, the samples were integrated and normalized using SCTransform^56^. Principal component analysis (PCA) was performed, and the top 10 components were retained based on the elbow point of the standard deviation plot for downstream analysis.

To better resolve the clonal architecture within each scRNA-seq sample, we used ClonoCluster v0.0.1^14^, an algorithm that integrates both transcriptomic profiles and clonal barcode identities into hybrid clusters. This approach replaced standard Louvain clustering and uniform manifold approximation and projection (UMAP) in the Seurat workflow, which did not produce optimal cluster separation due to the absence of clonal barcode information in its analysis. To fine-tune the impact of clonal barcode identity on the UMAP layout, we tested various Warp Factor (WF) values and determined that WF = 6 provided the best balance between transcriptomic similarity and clonal architecture.

### Clonal barcode classification and Shannon diversity analysis

To assess the clonal dynamics of barcoded cells over time, we calculated the Shannon diversity index^57^ for each sample based on the barcode abundance distributions. To classify individual barcode behaviors, we modeled their longitudinal abundance trajectories across all time points using nonlinear least squares fitting. Two competing models were tested for each barcode: an exponential decay model representing sensitive clones, and an exponential growth model representing persister clones. Barcodes best fit by the decay model (residual sum of squares, RSS < 0.1) were classified as Sensitive barcodes, whereas those better fit by the growth model (RSS < 0.1) were labeled as Persister barcodes. Barcodes that did not fit either model or remained at consistently low abundance were classified as multi-fate barcodes.

### Functional signature analysis by barcode group

To characterize the transcriptional programs and functional signatures associated with distinct clonal populations, we redefined the ClonoCluster-derived clusters into five barcode groups. Each group comprised a unique and mutually exclusive set of barcodes.

For each barcode group, we performed differential gene expression analysis to identify upregulated genes (log2 fold-change ≥ 1, detected in ≥ 50% of cells, and adjusted p-value < 0.05). These genes were subsequently submitted to the Enrichr platform for functional enrichment analysis^58^. Hallmark, KEGG, and Reactome databases were queried to determine biological processes associated with each group’s transcriptional program.

After defining the representative functional signatures for each group, we selected key genes from the enriched pathways and calculated signature activity scores using AUCell^59^ to validate the activity of these group-specific programs at single-cell level.

### SCENIC analysis

To infer the transcription factor activity and gene regulatory networks for each barcode group in the scRNA-seq data, we performed single-cell regulatory network inference and clustering (SCENIC) analysis using pySCENIC v0.12.1^59^. Gene co-expression modules were first inferred using the GRNBoost2 algorithm^60^. These modules were then refined by filtering target genes based on motif enrichment within ±10 kb of transcription start sites using the hg38 motif collection as a reference. Next, regulon activity was quantified across all cells using AUCell for each regulon. The resulting activity scores were integrated into Seurat objects to identify the top differentially active regulons across barcode types (Sensitive, Persister, and Multi-fate) and barcode groups defined from ClonoCluster.

### Alternative polyadenylation analysis

To investigate the post-transcriptional regulation associated with treatment response, we performed alternative polyadenylation analysis (APA) using MAAPER^61^ by comparing BAM files from drug treated samples to non-treated samples (day 21, day 57, endpoint vs. day 0; BRAFi/MEKi vs. CTRL). The required polyadenylation site (PAS) annotation file for hg38 was obtained from https://github.com/Vivianstats/data-pkg/tree/main/MAAPER/PolyA_DB. Genes with an adjusted p-value < 0.05 and log fold change > log (1.2) or < −log (1.2) were classified as lengthened or shortened, respectively.

### InferCNV analysis

To infer chromosomal copy number variations (CNVs) across barcode groups, we performed inferCNV v1.20.0^62^ on scRNA-seq data. The subcluster mode was used with both denoising and hidden Markov model (HMM) options enabled. The resulting CNV expression matrix was centered at 1. For each cell, a CNV score was calculated as the proportion of genes of which the inferred CNV values deviated from the expected range (0.9–1.1), defined as:

$$CNV_{\mathrm{score}}=\frac{1}{N}\sum_{i=1}^{N}\;I\left(x_{i}<0.9\right.\;or\;\left.x_{i}>1.1\right)$$

where x is the inferred CNV value for gene i, N is the total number of genes analyzed, and I is the indicator function.

### Computational analysis of spatial transcriptomics data

To examine the spatial organization of functional programs associated with barcode groups, particularly the intratumoral distribution of persister subpopulations, spatial transcriptomics was performed on a BRAFi/MEKi-treated endpoint tumor using the 10x Genomics Visium platform. The expression matrix and spatial coordinates were processed using Seurat v5, and spots with >20% reads mapped to the mouse genome were excluded.

As barcode information was not available in the Visium samples, each spot was annotated based on the functional signature with the highest AUCell enrichment score following normalization across all five signatures. For each annotated group, spatial autocorrelation was assessed using Moran’s Index statistic^63^. Cell-cell communication between groups was inferred using CellChat v2.1.2^64,65^ in the spatial mode.

### Computational analysis of bulk RNA-seq data

Raw sequencing data were aligned to the human reference genome (hg38) using STAR v2.7.11b^66^ and gene-level counts were quantified. Transcript abundance was normalized as transcript per million (TPM) for single-sample gene set enrichment analysis (ssGSEA)^67^. To assess temporal dynamics, the enrichment scores of the functional signatures identified in each barcode group were evaluated across time points.

### Analysis of acquired mutations

To assess the presence of acquired mutations following treatment, we applied the RNA_Mutect pipeline^68^ to bulk RNAseq data. Whole-exome sequencing (WES) was performed on matched normal peripheral blood mononuclear cell (PBMC) samples.

Identified variants were functionally annotated using ANNOVAR^69^, incorporating both the refGene and dbNSFP databases. This comprehensive annotation enabled the identification of potentially deleterious mutations by detecting variants absent in pre-treatment samples and recurrent across multiple post-treatment samples.

### Statistical tests

All statistical analyses were performed using R v4.4.0. Only post hoc false discovery rate (FDR) adjusted p-values less than 0.05 were considered significant.

## Supplementary Material

Supplement 1

Supplement 2

Supplementary Information is available for this paper.

## Data Availability

Raw and processed scRNA-seq, bulk RNA-seq, and CRISPR screening data were deposited in the Gene Expression Omnibus (GEO) under the accession number GSE299589. Raw and processed Visium spatial transcriptomic data are available under accession number GSE245582. Raw scRNA-seq data for the WM4380–2 model can be requested from Dr. Vito W. Rebebecca’s preprint paper^41^.

## Code availability

The analysis and visualization scripts for this study are available at GitHub (https://github.com/Yeqing95/MeRLin), along with a brief guide on reproducing the analysis workflows and figures presented in the paper.
