# Supplementary material for: Clonal dynamics shaped by diverse drug-tolerant persister states in melanoma resistance
Source: bioRxiv. 2025 Sep 19:2025.09.16.676608. Preprint. [Version 1] doi: 10.1101/2025.09.16.676608 (PMC12458950; doi:10.1101/2025.09.16.676608)
Supplement: Supplement 1 [file media-1.docx]

**
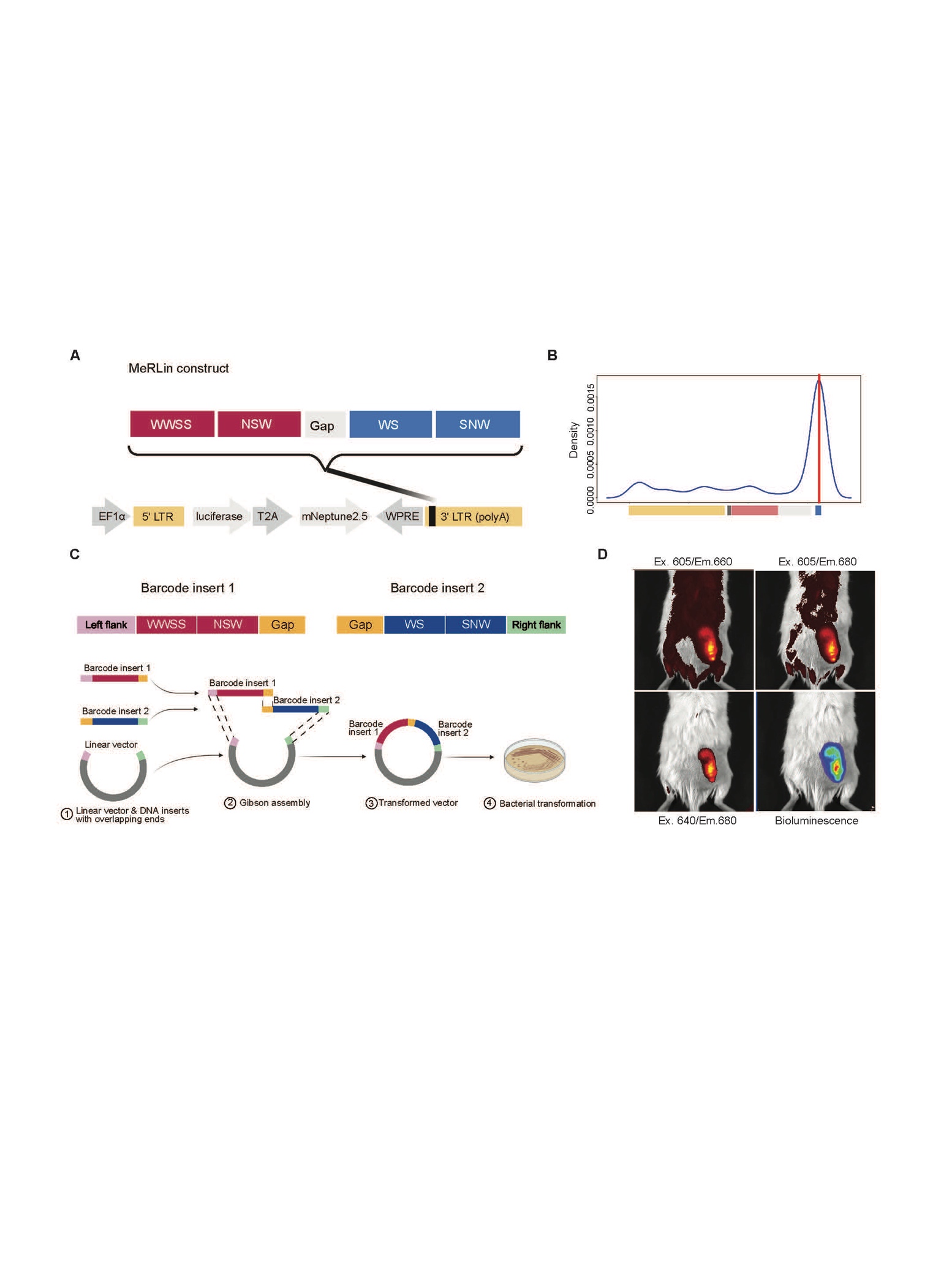
**

**Supplementary Figure 1. MeRLin vector optimized for barcode detection by scRNA-seq, RNA-FISH, and *in vivo* imaging.**

**a**, Schematic of the MeRLin vector design^13^. The vector encodes firefly luciferase and the far-red fluorescent protein mNeptune2.5, along with semi-random barcodes transcribed from the 3′ untranslated region (Methods). EF1α, elongation factor 1α; LTR, long terminal repeat; PolyA, polyadenylation signal; WPRE, woodchuck hepatitis virus post-transcriptional regulatory element. WWSS, NSW, WS, and SNW each repeated for 60 nucleotides (W = A or T; S = G or C; N = any base); Gap, sequence bridging the two barcode inserts (Supplementary Table 1). **b**, Coverage plot of scRNA-seq data showing the optimal barcode position in the vector for direct retrieval of barcode reads. Lentiviral components: yellow, luciferase; red, mNeptune2.5; blue, barcodes. **c**, Gibson assembly was used to construct the MeRLin plasmid library^13^ (Methods and Supplementary Table 1). The resulting 265 bp barcodes enable visualization of specific cancer subpopulations via targeted RNA fluorescence *in situ* hybridization (RNA-FISH) (Methods). **d**, *In vivo* imaging (IVIS Spectrum) of a mouse bearing a MeRLin-barcoded WM4237-1 tumor using bioluminescence and mNeptune2.5 fluorescence with far-red excitation and emission settings optimized for tissue penetration.


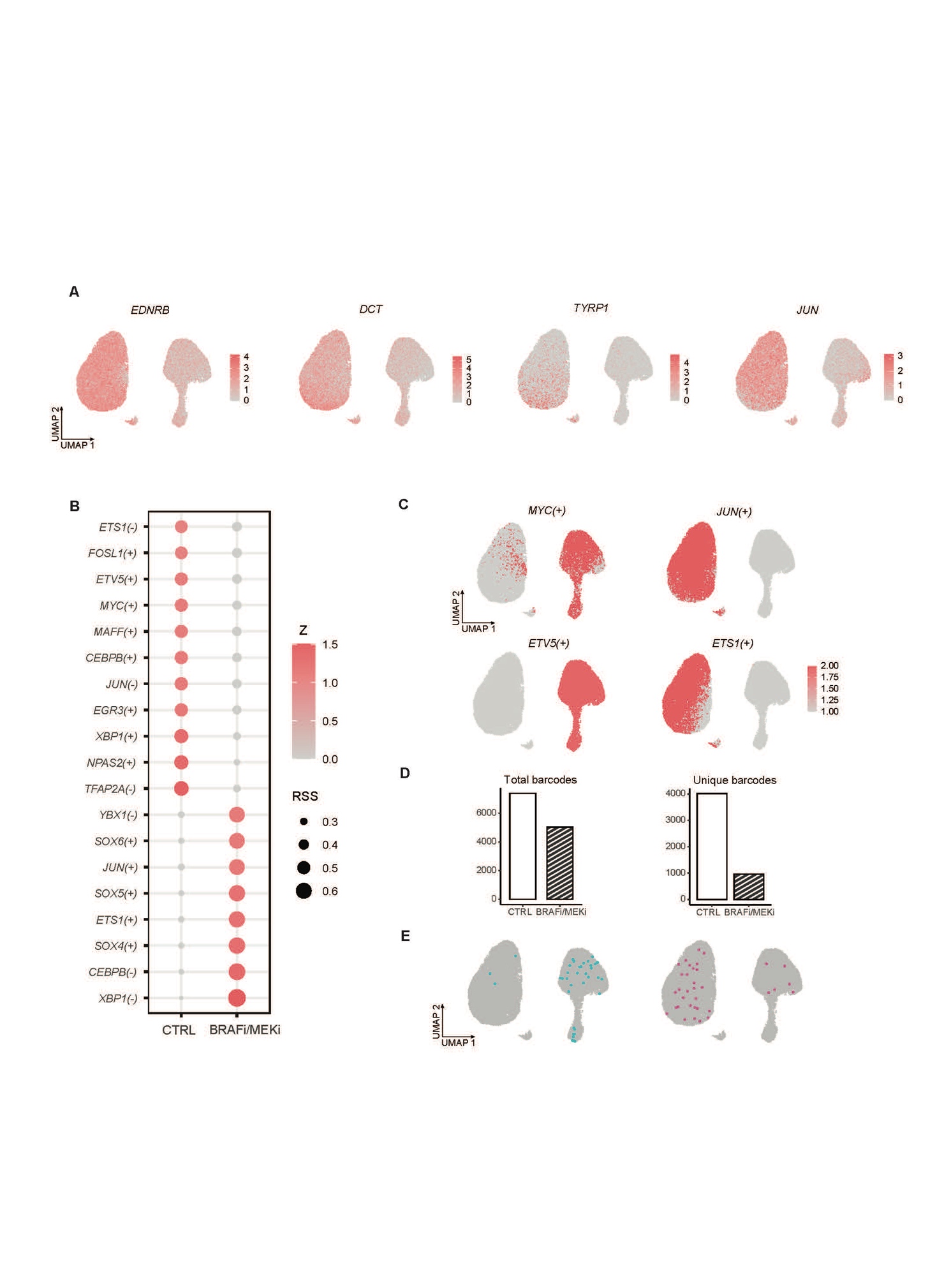


**Supplementary Figure 2. Transcriptional and clonal changes in WM4237-1 cells following BRAFi/MEKi treatment.**

**a**, UMAP showing expression of melanocytic genes (*EDNRB, DCT, TYRP1*), and the transcription factor *JUN*, which are differentially expressed in BRAFi/MEKi-treated cells compared to control. **b**, SCENIC analysis identifying key regulators from scRNA-seq data. **c,** Regulon activities of transcription factors *MYC*, *JUN*, *ETV5* and *ETS1*. **d**, Total barcode numbers and unique barcode numbers measured following BRAFi/MEKi treatment. **e,** UMAP showing a representative sensitive clone (blue) and a resistant clone (red).


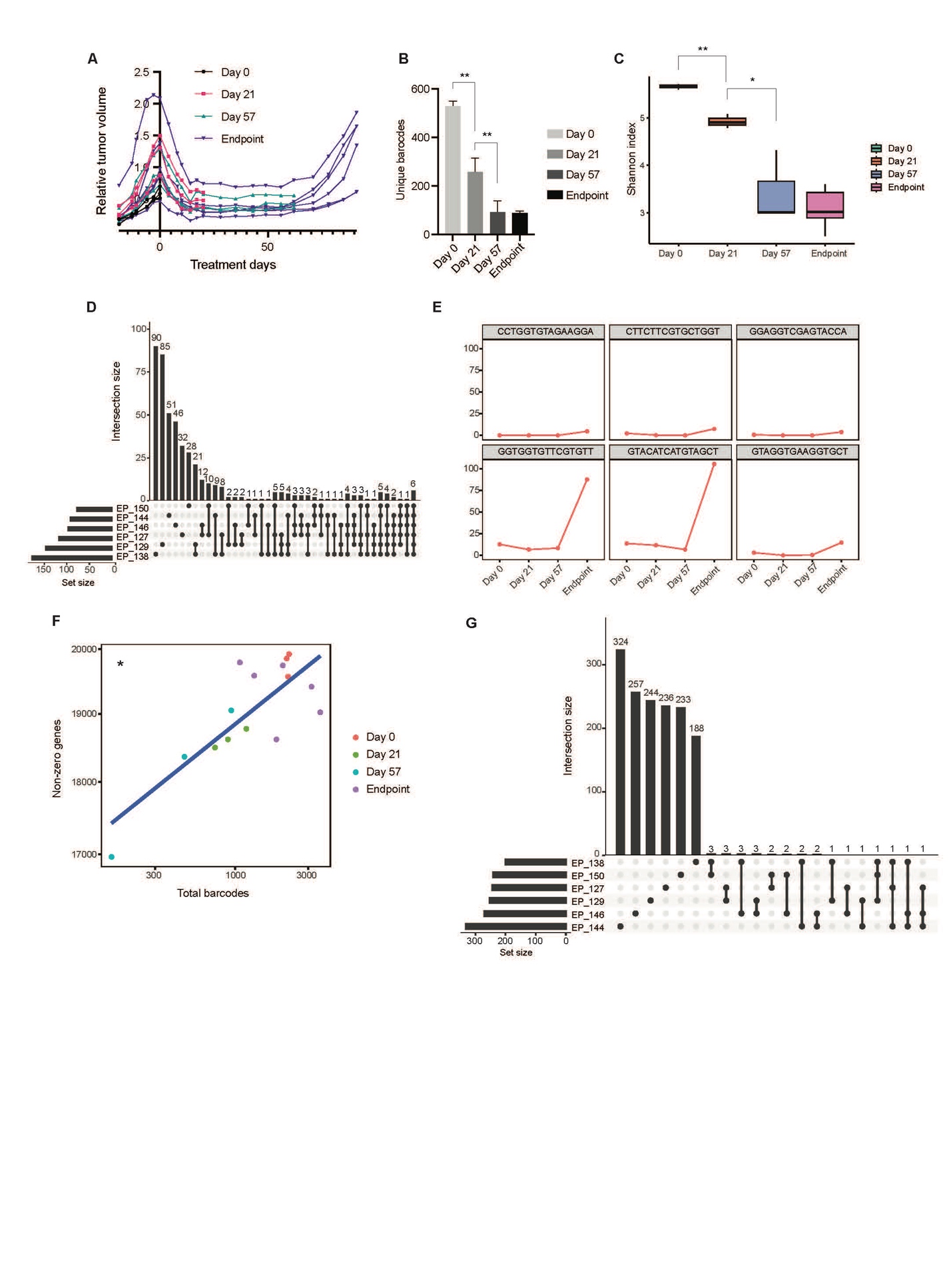


**Supplementary Figure 3. Barcode distribution across BRAFi/MEKi-treated WM4237-1 tumors based on bulk RNA-seq data.**

**a,** Growth curves of individual MeRLin-barcoded WM4237-1 tumors during BRAFi/MEKi treatment. Tumor harvest time points are indicated by four colors. **b,** Number of unique barcodes retrieved from each time point. **P < 0.01; one-tailed t-test. **c**, Shannon diversity indices revealed a moderate decrease in barcode diversity by day 21, followed by a significant reduction at day 57. *P < 0.05, **P < 0.01; one-tailed t-test. **d,** Plot illustrating shared barcodes among endpoint (EP) replicate tumors. **e,** Normalized abundance of each of the six shared barcodes across treatment time points, as identified in panel **d**. **f,** Plots depicting global transcriptional activity across time points for genes and barcodes with non-zero expression. Pearson's correlation, r = 0.58, P = 0.025. **g,** RNA-MuTect analysis revealed no acquired mutations are shared across the six resistant endpoint (EP) tumors, compared to pre-treatment tumors.


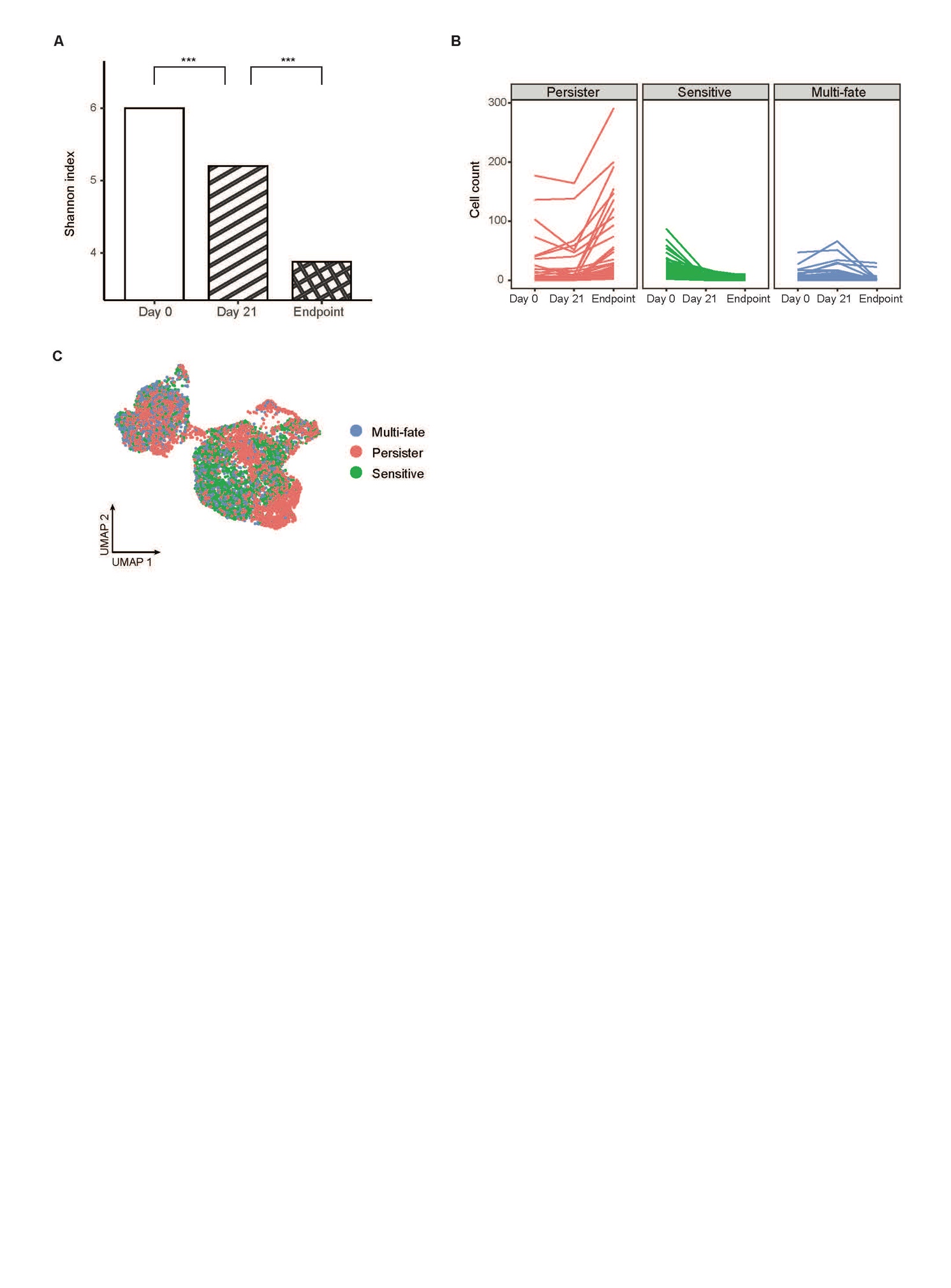


**Supplementary Figure 4. Tracking clonal and transcriptional states in BRAFi/MEKi-treated WM4237-1 tumors using scRNA-seq.**

**a,** Shannon diversity indices showed a significant decrease in barcode diversity by day 21, followed by a further reduction at the resistant endpoint. ***P < 0.001; two-tailed Hutcheson t-test. **b,** Clonal fates, determined by the normalized abundance of individual barcodes during treatment, are categorized as persister (red), sensitive (green), and multi-fate (blue). **c,** UMAP visualization of clonal subpopulations across three treatment time points, highlighting persister (red), sensitive (green), and multi-fate (blue) cells.


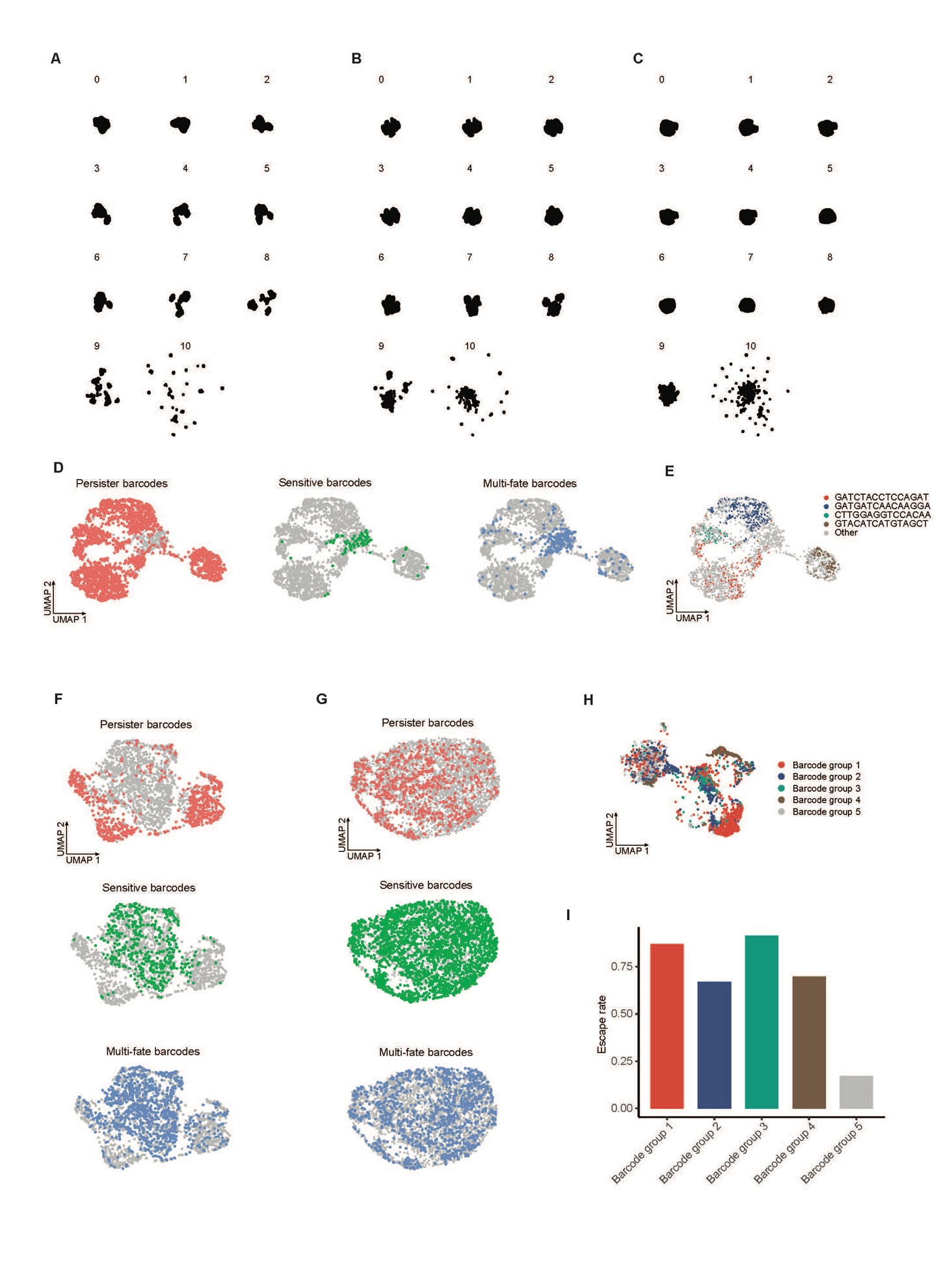


**Supplementary Figure 5. ClonoCluster integrates clonal and transcriptomic information to reveal distinct clonal fates.**

**a**, Effect of increasing Warp Factor value on UMAP structure for scRNA-seq data of WM4237-1 endpoint tumors, day 21 tumors (**b**), and day 0 tumors (**c**). **d**, UMAP visualizations of hybrid clusters showing their associated clonal fates including persister (red), sensitive (green), and multi-fate (blue) subpopulations. **e**, UMAPs displaying the expression patterns of the most dominant barcode from barcode group 1 (red), 2 (blue), 3 (green), and 4 (brown). **f.** UMAPs showing persister (red), sensitive (green), and multi-fate (blue) subpopulations in WM4237-1 day 21 tumors. **g.** UMAPs illustrating persister (red), sensitive (green), and multi-fate (blue) subpopulations in WM4237-1 day 0 tumors. **h,** UMAP visualization of 5 barcode groups from the endpoint tumor. **i**, Proportions of each endpoint barcode group escaping the non-proliferative MRD state and re-entering a proliferative state resembling the pre-treatment tumor.

**
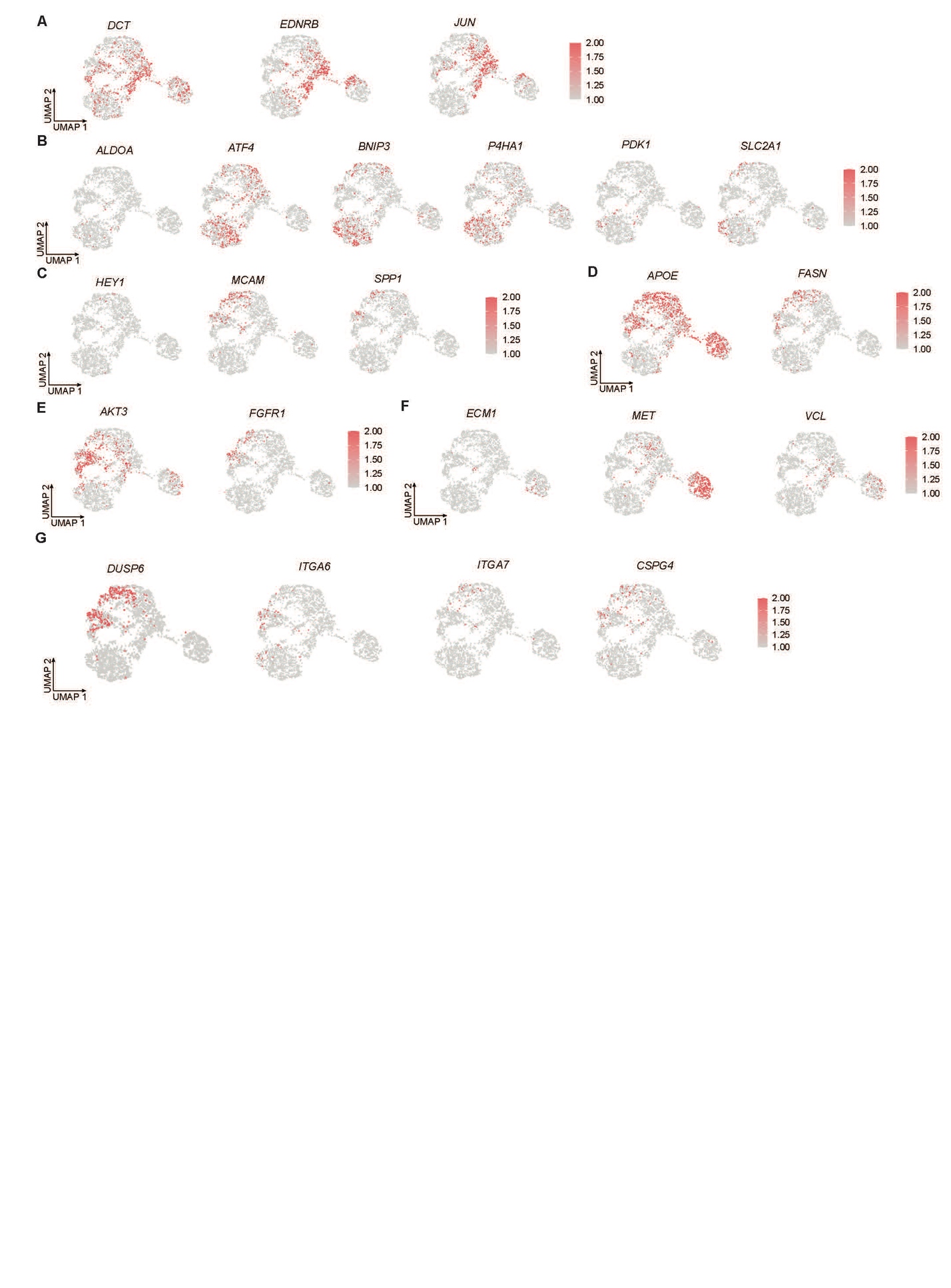
**

**Supplementary Figure 6. Key genes characterizing each persister state.**

**a**, UMAP showing expression of melanocytic markers *DCT, EDNRB,* and *JUN*, stress-like genes *ALDOA*, *ATF4*, *BNIP3*, *P4HA1*, *PDK1*, and *SLC2A1* (**b**), NC-like genes *HEY1*, *MCAM*, and *SPP1* (**c**), lipid metabolism genes *APOE* and *FASN* (**d**), PI3K signaling genes *AKT3* and *FGFR1* (**e**), and ECM remodeling genes *ECM1*, *MET* and *VCL* (**f**). **g**, UMAPs showing *DUSP6*, *ITGA6*, *ITGA7*, and *CSPG4* expression within barcoded WM4237-1 persister subpopulations.


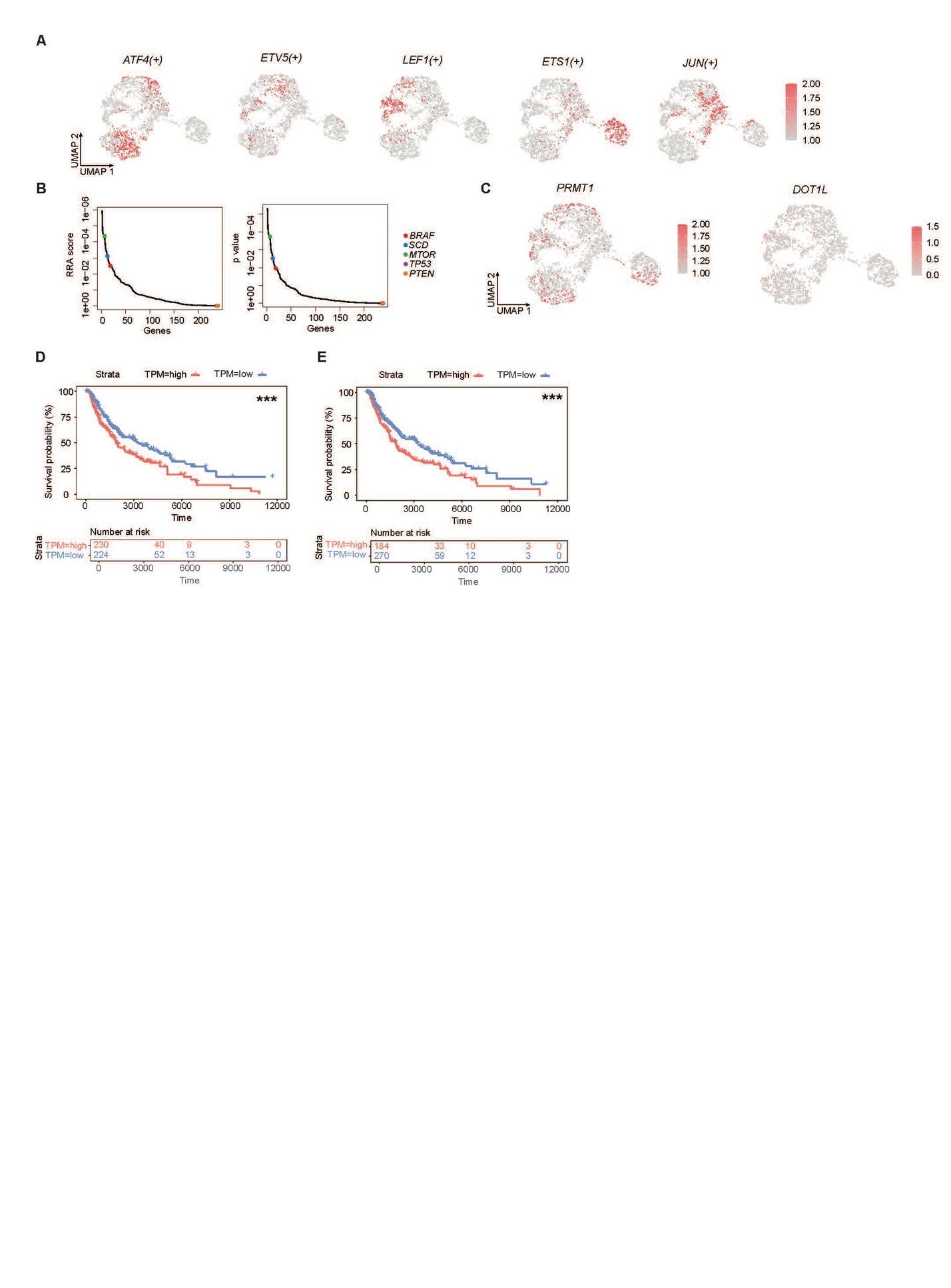


**Supplementary Figure 7. Transcriptional factors and epigenetic regulators underlying melanoma resistance.**

**a,** SCENIC analysis showing regulon activity of transcription factors *ATF4* for the stress-like, *ETV5* for lipid metabolism, *LEF1* for PI3K signaling, *ETS1* for ECM remodeling, and *JUN* for melanocytic state. **b**, Low negative RRA (Robust Rank Aggregation) scores and p-values for *MTOR*, *SCD*, and *BRAF* indicate strong depletion upon knockout, whereas *PTEN* and *TP53* knockouts are among the most enriched phenotypes. **c**, UMAPs illustrating *PRMT1* and *DOT1L* expression within barcoded WM4237-1 persister subpopulations. **d**, Survival analysis of TCGA data revealed that high expression of *PRMT1* (P = 0.0014) and (**e**) *DOT1L* (P = 0.0027) is associated with poorer patient outcomes.


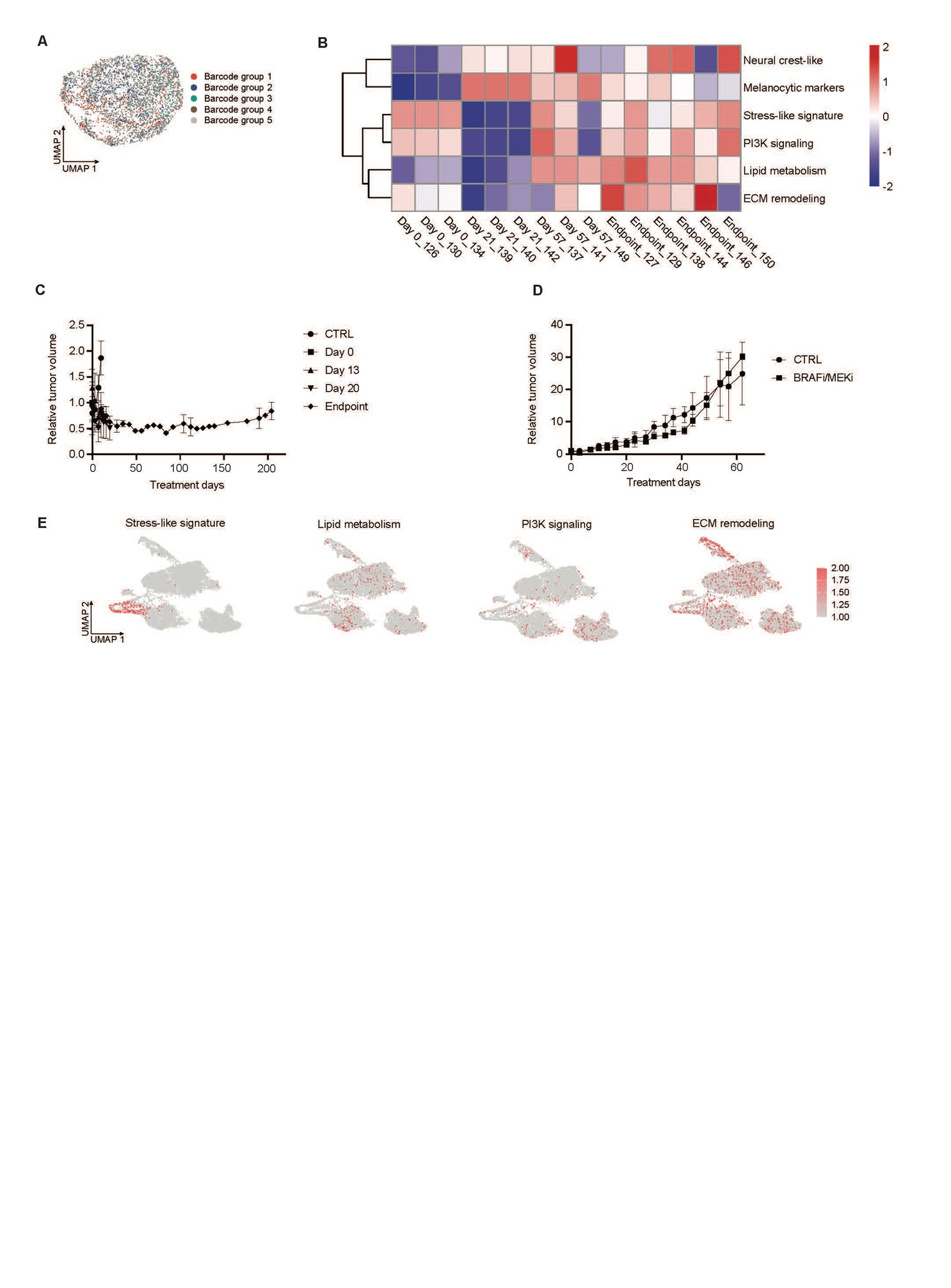


**Supplementary Figure 8. Diverse drug-tolerant persister states arise throughout treatment and are shared across PDX models.**

**a**, ClonoCluster applied to scRNA-seq data from day 0 barcoded WM4237-1 tumors. UMAPs show barcode groups 1-5 (red, blue, green, brown, gray) from endpoint tumors. **b**, Heatmap showing persister gene signatures derived from bulk RNA-seq of barcoded WM4237-1 tumors across BRAFi/MEKi treatment. **c**, WM4007 initially responded to BRAFi/MEKi treatment but relapsed after approximately seven months. **d**, WM4380-2 exhibited strong resistance to BRAFi/MEKi treatment. **e**, UMAPs showing persister signatures from non-barcoded WM4007 scRNA-seq data at the MRD stage on day 20.


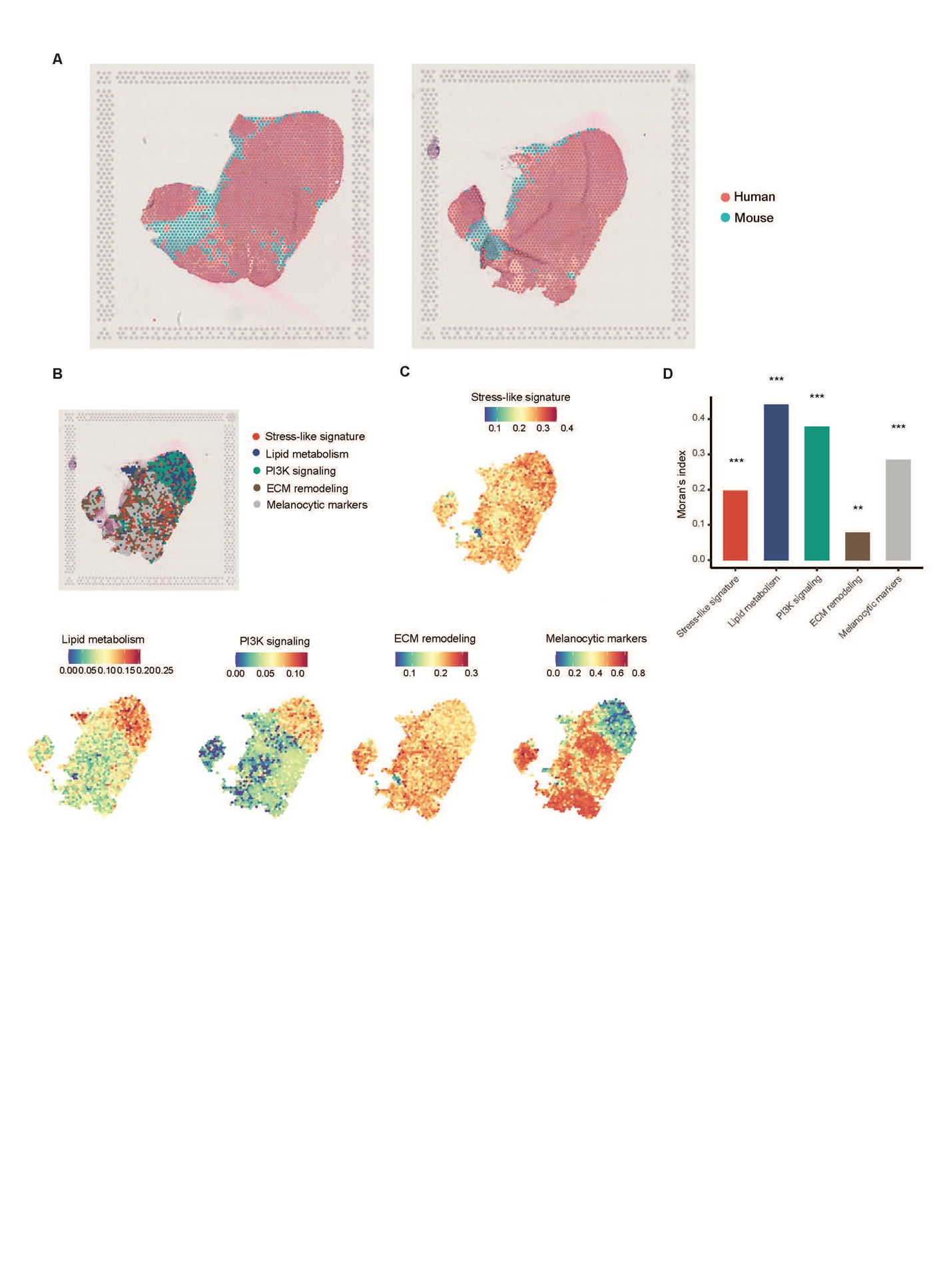


**Supplementary Figure 9. Spatial mapping of persister states in barcoded WM4237-1 tumor.**

**a**, Spatial transcriptomics showing human tumor and mouse stromal reads on two replicate slides from the WM4237-1 endpoint tumor. **b**, Spatial transcriptomics showing persister states overlaid on H&E-stained section of a recurrent WM4237-1 PDX tumor. Stress-like (red), lipid metabolism (blue), PI3K signaling (green), and ECM remodeling (brown) and melanocytic (grey) states. **c**, Distinct distribution of each persister state, colored as in **b**. **d**, Moran's indices showing spatial autocorrelation for each persister state. Stress-like (red), I = 0.20; lipid metabolism (blue), I = 0.44; PI3K signaling (green), I = 0.38; ECM remodeling (brown), I = 0.078; melanocytic (grey), I = 0.28. **P < 0.01, ***P < 0.001.


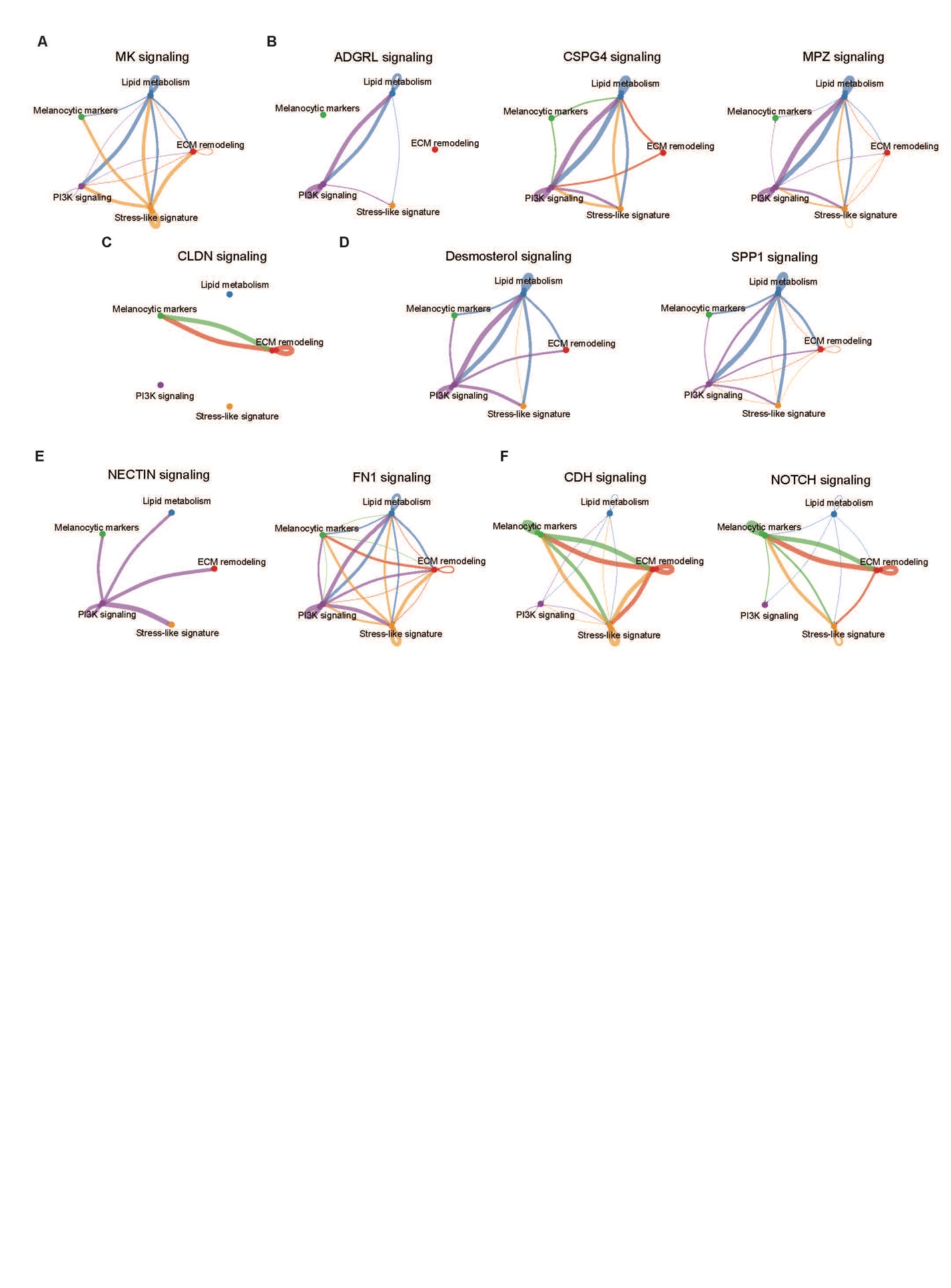


**Supplementary Figure 10. CellChat analysis revealed the strength of cell-cell communications among persister states.**

**a**, Stress-like (yellow) and lipid metabolism (blue) state emitted Midkine (*MK*) signals. **b**, Lipid metabolism (blue) and PI3K signaling (purple) states exhibited ANGPTL, CSPG4, and MPZ signaling. **c**, ECM remodeling state (red) sent Claudin (*CLDN*) signaling. **d**, Lipid metabolism state (blue) showed Desmosterol and osteopontin (*SPP1*) signaling. **e**, PI3K signaling state (purple) emitted Nectin and Fibronectin 1(*FN1*) signaling. **f**, Notch signaling (right) exhibited a similar pattern to cadherin (*CDH*) signaling (left).
